# GM-CSF downregulates type I IFN responses in glioblastoma-associated monocytes

**DOI:** 10.1038/s12276-026-01769-1

**Published:** 2026-07-03

**Authors:** Sofie P. Meyer, Rebekka Bauer, Michelle Weidmann, Blerina Aliraj, Megan A. Palmer, Evelyn Sirait-Fischer, Igor Mačinković, Aneta Buczynska, Irina Bode, Giulia Cardamone, Melanie Flohr, Katharina J. Weber, Andreas Weigert, Bernhard Brüne, Tobias Schmid

**Affiliations:** 1 Institute of Biochemistry I, Faculty of Medicine, Goethe University Frankfurt, Frankfurt am Main, Germany; 2 Neurological Institute (Edinger Institute), University Hospital, Goethe University Frankfurt, Frankfurt am Main, Germany; 3 Frankfurt Cancer Institute, Frankfurt am Main, Germany; 4 University Cancer Center (UCT), University Hospital, Goethe University Frankfurt, Frankfurt am Main, Germany; 5 German Cancer Consortium (DKTK), Partner Site Frankfurt, and German Cancer Research Center (DKFZ), Heidelberg, Germany

**Keywords:** Tumour immunology, Cancer microenvironment

## Abstract

Glioblastoma (GB) is the most aggressive type of brain cancer with a devastating prognosis. Myeloid cells, especially monocytes and macrophages, comprise the majority of immune cells within the GB tumour microenvironment, where they contribute to the development of an immunosuppressive milieu hindering potential anti-tumour immune responses. Type I IFNs usually facilitate a pro-inflammatory shift in monocytes and macrophages and allow for anti-tumour functions. Using RNA sequencing, we observed that type I IFN-stimulated genes (ISGs) are globally downregulated in primary human monocytes, but not microglia, co-cultured with GB cells both upon direct cell–cell contact and upon the exchange of soluble factors. Surprisingly, the reduction of type I IFN responses did not result from changes in interferon-α/β-receptor availability and activation. Instead, we identified GM-CSF as a critical, GB cell-derived factor, which decreased ISG expression in monocytes at extremely low concentrations via induction of the TGF-β signalling pathway. In line, type I IFN response gene expression and GM-CSF activity in human GB biopsies appeared to be mutually exclusive. Specifically, active GM-CSF signalling appeared to inhibit ISG expression in the same cell as well as in neighbouring monocyte-derived macrophages, which resulted in lower T cell recruitment. In conclusion, our data provide evidence for a so far unknown GM-CSF- and TGF-β-dependent type I IFN inhibitory mechanism in GB with potential implications for future therapeutic approaches.

## GM-CSF Inhibits Type I IFN Responses in Glioblastoma

Glioblastoma (GB) is a highly aggressive brain cancer with a poor prognosis, characterized by a complex tumour microenvironment that includes various immune cells. This study investigates the regulation of type I IFN responses in monocytes interacting with GB cells, revealing a significant reduction in interferon-stimulated gene (ISG) expression. Researchers co-cultured primary human monocytes with GB cell lines and found that GM-CSF secreted by GB cells plays a crucial role in downregulating ISG expression, partly through TGF-β signalling. Importantly, this downregulation correlates with reduced T cell recruitment in patient tumours, highlighting its impact on anti-tumour immunity. The findings suggest that GM-CSF and TGF-β are key players in modulating immune responses in the GB tumour microenvironment, offering potential targets for therapeutic intervention. Future research could explore the interplay between hypoxia and ISG expression in the GB context.

This summary was initially drafted using artificial intelligence, then revised and fact-checked by the author.

## Introduction

Glioblastoma (GB) is a grade 4 diffuse astrocytic tumour without isocitrate dehydrogenase or histone 3 mutations (IDH- and H3-wildtype) according to the 2021 WHO classification of central nervous system tumours, and the most aggressive type of brain cancer in adults^1^. GB bears a poor prognosis with a median overall survival of 15 months for patients receiving standard therapy, i.e. radiotherapy plus concomitant and adjuvant chemotherapy with temozolomide^2^. GB is characterized, among others, by microvascular proliferation and necrosis^1^, intratumoural genetic and transcriptomic heterogeneity^3^ and hypoxia^4^. The GB tumour microenvironment (TME) contains numerous non-cancerous cell types, including endothelial cells, pericytes, fibroblasts, astrocytes, neurons and immune cells^5^. Immunologically, the brain is considered to be a specialized niche owing to the blood–brain barrier (BBB), which restricts access of peripheral immune cells, but also because of distinct resident immune-cell populations, especially microglia^6^. However, in GB, the BBB is often leaky, allowing for infiltration of immune cells, predominantly myeloid cells, which establish an immunosuppressive milieu^5^. Consequently, tumour-associated macrophages (TAMs) constitute the majority of immune cells (commonly more than 75%) in the GB TME, whereas the number of lymphocytes is usually very low^7–9^. Macrophages in the homeostatic brain are largely yolk sac-derived microglia and border-associated macrophages. The latter reside in meninges, choroid plexus or the perivascular space, and originate from and are replenished by either yolk sac cells or circulating monocytes, depending on the subtype^10,11^. Accordingly, TAMs in the GB TME either originate from brain-resident macrophages or differentiate from infiltrating peripheral blood monocytes. Although the discrimination of the different TAM subtypes is still challenging, new technologies, including single-cell or spatial transcriptomics, identified novel markers (e.g. CD49d) allowing for detailed and unbiased insights into the composition and functional differences of TAM populations within the GB TME^8,9,12^. Importantly, many TAM subtypes in GB share an immunosuppressive phenotype, contributing to a widespread failure of immunotherapeutic approaches in GB^13,14^. TAM polarization in GB is predominantly controlled by secreted factors from tumour cells, and TAMs in turn secrete various factors, thereby suppressing anti-tumour immune responses, but also directly promoting tumour growth and invasion^15–19^.

One crucial inflammatory pathway for functional myeloid anti-tumour immunity is the type I interferon (IFN) signalling axis^20^. Type I IFNs were initially discovered in the context of viral infections, making them important mediators during anti-viral immunity^21,22^. When type I IFNs are synthesized, they are readily secreted and elicit specific responses by binding to the interferon-α/β-receptor (IFNAR), which activates downstream signalling via Janus kinase 1 (JAK1) and signal transducer and activator of transcription 1 (STAT1), eventually facilitating the expression of interferon-stimulated genes (ISGs)^23,24^. In the tumour context, type I IFNs are intrinsically generated, e.g. owing to the presence of self-nucleic acids, and are massively induced upon immunogenic cell death after chemo- or radiotherapy^25,26^. Exposure of TAMs to type I IFNs subsequently elicits anti-tumour immune responses by inducing pro-inflammatory polarization, enhancing tumour cell killing, and shaping a tumour-suppressive TME, e.g. via the promotion of T cell recruitment^20^. Interestingly though, little is known about the regulation of type I IFN responses in TAMs in GB.

We therefore aimed to elucidate how type I IFN responses are regulated in primary human monocytes during their communication with GB cells and observed a global downregulation of ISG expression in monocytes co-cultured with GB cells, owing to GB-secreted granulocyte-macrophage colony-stimulating factor (GM-CSF), a response involving transforming growth factor-β (TGF-β) signalling. The in vitro findings were further supported in tumour sections from GB patients by the mutually exclusive expression of ISGs and a marker for GM-CSF activity, as well as by the higher distance of monocyte-derived (MO)-TAMs with high ISG expression compared with those expressing low ISG levels from cells displaying high GM-CSF activity. Lower ISG expression in MO-TAMs further correlated with reduced T cell recruitment in primary GB samples.

## Materials and Methods

### Chemicals

Chemicals came from Sigma-Aldrich (Taufkirchen, Germany), if not indicated otherwise (details see Supplementary Table 1).

### Cell culture

Peripheral blood mononuclear cells (PBMCs) were isolated from human buffy coats of anonymous healthy donors, provided by DRK Blutspendedienst Baden-Württemberg-Hessen (Frankfurt, Germany) using Pancoll gradients (PAN Biotech, Aidenbach, Germany). Depletion of erythrocytes was performed by incubating PBMCs for 20 s in distilled water and adding a 0.6 M potassium chloride solution. Primary human monocytes were isolated from PBMCs using magnetic activated cell sorting (MACS) with CD14 MicroBeads (Miltenyi Biotec, Bergisch Gladbach, Germany). Monocytes were cultured in Roswell Park Memorial Institute (RPMI) 1640 medium, supplemented with 100 U/ml penicillin, 100 µg/ml streptomycin, 10% fetal bovine serum (FBS) (Capricorn Scientific GmbH, Ebsdorfergrund, Germany) and 50 ng/ml recombinant human macrophage colony-stimulating factor (M-CSF). The human C20 MG cell line was kindly provided by Dr. David Alvarez-Carbonell (Department of Molecular Biology and Microbiology, Case Western Reserve University, Cleveland, OH, USA)^27^. The human microglial clone 3 cell line HMC3 and tumour cell lines were obtained from ATCC-LGC Standards GmbH (Wesel, Germany). Tumour cells were cultured in RPMI 1640 medium supplemented with 100 U/ml penicillin, 100 µg/ml streptomycin and 10% FBS. Microglial cell lines C20 and HMC3 were cultured in Dulbecco’s modified eagle medium (DMEM) supplemented with 100 U/ml penicillin, 100 µg/ml streptomycin and 10% FBS^28^. All cells were cultured at 37°C and 5% CO_2_ in a humidified atmosphere. Media were purchased from Thermo Fisher Scientific (Dreieich, Germany).

### Co-culture

For direct co-culture experiments, tumour cells were seeded into culture dishes (Greiner Bio-One, Frickenhausen, Germany). After 24 h, medium was removed and monocytes were placed on top of tumour cells. Monocytes were separated from tumour cells by fluorescence-activated cell sorting (FACS) based on the expression of CD45, CD14 and CD16, and the absence of CD133. For indirect co-culture experiments, monocytes or microglia were seeded into culture dishes and tumour cells were seeded into transwells (0.4 µm, Sarstedt, Nümbrecht, Germany) 24 h before and placed on top of the monocytes or microglia.

### Flow cytometry and cell sorting

For flow cytometry and FACS, supernatants from cultures containing cells in suspension were collected and adherent cells were detached carefully with a cell scraper on ice. After centrifugation and washing in PBS with 0.5% bovine serum albumin (BSA), 2% human FcR Blocking Reagent (Miltenyi Biotec) was added for 10 min on ice to block non-specific antibody binding to Fc-γ receptors. Single-cell suspensions were stained with fluorochrome-coupled antibodies listed in Supplementary Table 2 and combined in Brilliant Stain buffer (BD Biosciences, Heidelberg, Germany) for 20 min on ice. Cells were analysed by flow cytometry using a FACSymphony™ A5SE flow cytometer or sorted using a FACSymphony™ S6 cell sorter (BD Biosciences). Data analysis was performed with FlowJo software V10 (Tree Star, Ashland, OR, USA). CompBeads (BD Biosciences) were used to create multicolour compensation matrices for single-colour compensation.

### Cytokine quantification

Supernatants of mono- and co-cultures and different tumour cell lines were centrifuged at 500 x *g* for 5 min and snap-frozen. For the measurement of cytokine concentrations, cell-culture supernatants were thawed and centrifuged at full speed for 5 min. Interleukin (IL)-6, IL-1β, IL-10 and TGF-β quantities were assessed with cytometric bead array (CBA) flex sets (BD Biosciences) according to the manufacturer’s instructions. IFN-α2, thymic stromal lymphopoietin (TSLP), IL-1α, IL-1β, GM-CSF, IL-11, IL-12p40, IL-12p70, IL-15, IL-18, IL-23, IL-27 and IL-33 were quantified using the LEGENDplex™ Human Cytokine Panel 2 (BioLegend, Koblenz, Germany) according to the manufacturer’s instructions. Samples were acquired with a FACSymphony™ A5SE flow cytometer. GM-CSF concentrations were quantified using the Human GM-CSF Quantikine HS ELISA Kit (Biotechne, Wiesbaden, Germany), according to the manufacturer’s instructions for serum/plasma samples. The measurements were performed on a Tecan spark plate reader (Tecan, Crailsheim, Germany).

### Western blot

For protein analyses, monocytes in suspension and adherent cells were harvested, centrifuged at 500 x *g* and 4°C for 5 min, and lysed in lysis buffer (10 mM Tris-HCl, 6.65 M Urea, 10% glycerol, 1% sodium dodecyl sulfate (SDS) (Carl Roth, Karlsruhe, Germany), pH 7.4, freshly supplemented with 1 mM dithiothreitol (DTT) (Carl Roth), cOmplete protease inhibitor and phosSTOP phosphatase inhibitor mixes (both Roche, Grenzach-Wyhlen, Germany)). After sonication, protein concentration was determined by a *DC* protein assay kit (Bio-Rad, Munich, Germany). The separation of 60–80 µg total protein was performed by SDS-polyacrylamide gel electrophoresis (SDS-PAGE), and proteins were transferred onto 0.2- or 0.45-µm nitrocellulose membranes (GE Healthcare, Solingen, Germany) using a Trans Blot Turbo blotting system (Bio-Rad). Membranes were stained with the Revert 700 Total Protein Stain kit (LI-COR Biosciences, Bad Homburg, Germany) according to the manufacturer’s instructions and blocked in 5% BSA or 5% milk in tris-buffered saline with Tween-20. Proteins were detected using specific antibodies (Supplementary Table 3) and appropriate IRDye secondary antibodies (LI-COR Biosciences) on an Odyssey infrared imaging system and quantified with Image Studio Digits 5.0 software (both LI-COR Biosciences).

### RNA isolation, reverse transcription and quantitative polymerase chain reaction

Total RNA was isolated using either TRIzol (Thermo Fisher Scientific) or RNeasy Mini or Micro Kits (Qiagen, Hilden, Germany) according to the manufacturers’ instructions. Reverse transcription was performed with the Maxima First Strand cDNA Synthesis Kit and PowerUp SYBR Green Master Mix was used on the QuantStudio 3/5 Real-Time PCR System (all Thermo Fisher Scientific) to perform qPCR analyses. Primers were obtained from Biomers (Ulm, Germany) and are listed in Supplementary Table 4.

### RNA sequencing

RNA quality and quantity were assessed using RNA ScreenTape assays on a TapeStation 4150 (Agilent Technologies, Waldbronn, Germany) and Qubit RNA HS Assay Kits on a Qubit 3.0 Fluorometer (Thermo Fisher Scientific). Sequencing libraries were prepared with the QuantSeq 3’mRNA-Seq V2 Library Prep Kit FWD with UDI (12 nt) and the PCR Add-on Kit for Illumina (Lexogen, Vienna, Austria). Library quality was measured using HS-D1000 ScreenTape assays, and library quantities were determined with Qubit dsDNA HS Assay Kits. Sequencing of 0.75-nM libraries (single-end, 75 cycles) was performed on a NextSeq2000 platform using NextSeq 1000/2000 P2 Reagents (100 cycles) v3 with 2% spike-in of PhiX Control v3 (all Illumina, San Diego, CA, USA). For details on the bioinformatics analyses of RNA and scRNA sequencing data (GSM4972211, ND1-7)^9^ see Supplementary Information.

### Multiplex fluorescent immunohistochemistry

Tissue microarrays from 40 patients with GB were provided by the UCT biobank. The study was approved by the Ethical Committee at the University Hospital Frankfurt (ethics vote: SNO-2-2023). Tissues were stained by multiplex fluorescent immunohistochemistry (mfIHC) staining using OpalTM 7-Color Fluorescent IHC Kits (Akoya, Marlborough, MA, USA). The antibodies listed in Supplementary Table 5 were used and automatically stained with the BOND RX Automated IHC Research Stainer (Leica Biosystems, Nussloch, Germany). Stained tissue microarrays were scanned with the Vectra® 3 automated quantitative pathology imaging system (Akoya). For further details on the bioinformatics analyses of the tissue microarrays see Supplementary Information.

### Statistical analysis

Statistical analyses were carried out with GraphPad Prism v10.5.0 (GraphPad Software, San Diego, CA, USA). Data are presented as means ± SEM of at least three independent experiments. Data from IHC are presented as boxplots depicting median, interquartile range and minimum/maximum. For detailed information on the statistical tests used, see Supplementary Information.

## Results

### Type I IFN-stimulated genes are differentially expressed in tumour-associated monocytes and macrophages in the glioblastoma tumour microenvironment

**Fig. 1:**
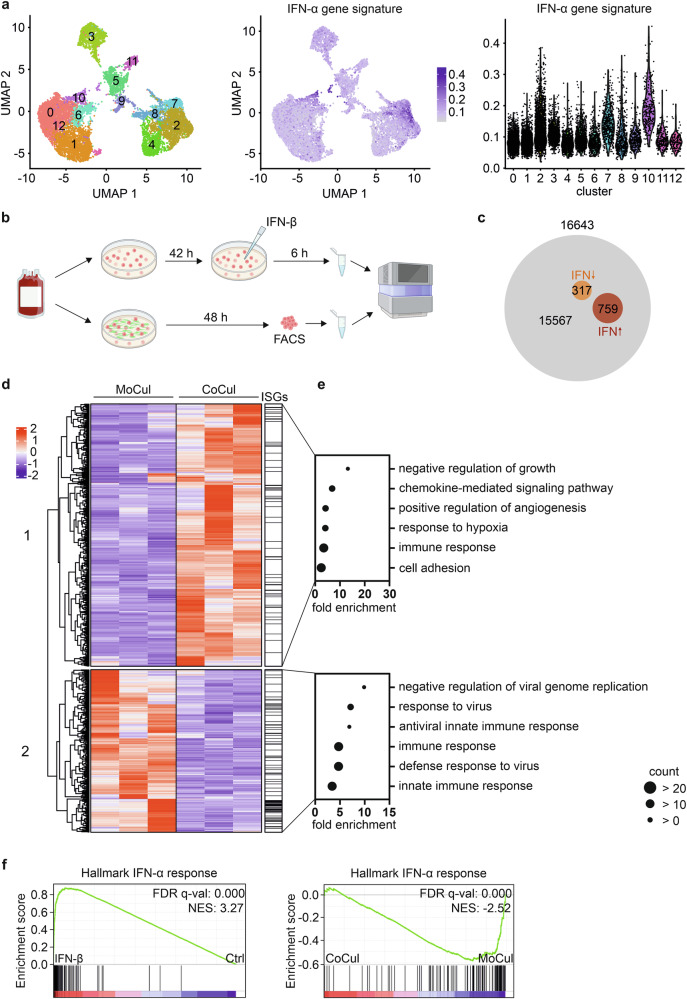
Expression of type I IFN response genes in tumour-associated myeloid cells in the glioblastoma tumour microenvironment. **a**, Uniform manifold approximation and projection (UMAP) of CD45-positive cells with substantial expression of myeloid markers from seven patients with newly diagnosed GB^9^ (*left panel*) and relative expression of an interferon (IFN)-α gene signature scored with UCell visualized as feature (middle panel) and violin plots (right panel). **b**, Experimental procedure of the in vitro experiments: primary human monocytes were isolated from buffy coats using CD14-MACS, before being cultured for 48 h either in mono-culture (MoCul) or in co-culture (CoCul) with LN229 cells. Alternatively, monocytes were treated with 1 ng/ml IFN-β for the last 6 h. After fluorescence-activated cell sorting (FACS)-based purification, RNA was isolated form monocytes and RNA expression was determined by RNA sequencing (*n* = 3). **c**, Venn diagram depicting all genes captured by RNA-seq in monocytes, and highlighting subgroups of genes up- or downregulated (*padj* < 0.1, |log2FC | > 1) after IFN-β stimulation. **d**, Differentially expressed genes (*z*-score normalized counts) between mono- and co-cultured monocytes (*padj* < 0.1, |log2FC | > 1), categorized by *k*-means clustering. Annotation column depicts genes upregulated in mono-cultured monocytes treated with IFN-β (*padj* < 0.1, log2FC > 1) from **c**, herein determined as interferon-stimulated genes (ISGs). **e**, Top six functional annotation terms for each cluster identified by the Database for Annotation, Visualization and Integrated Discovery, ordered by fold enrichment (size of circles represents counts of enriched genes within the annotation clusters). **f**, Gene set enrichment analysis of untreated versus IFN-β-treated monocytes (left panel), or mono- versus co-cultured monocytes (right panel). FDR, false discovery rate; NES, normalized enrichment score.

Macrophages, which comprise the majority of the immune infiltrate in GB, exert various anti-tumour actions when exposed to type I IFNs^20^. However, surprisingly little is known about type I IFN signatures in GB-associated macrophages. Therefore, we re-analysed scRNA sequencing data of seven patients with newly diagnosed GB from a previous study (GSM4972211)^9^, focusing on CD45-expressing cells to specifically investigate immune-cell subsets. Clustering analysis of the immune-cell subtypes identified 14 distinct cluster populations, the majority of which contained cells of myeloid origin (Supplementary Fig. 1a and b). Specifically, while markers for innate and adaptive lymphocytes (*CD3G, granzyme A (GZMA)* and *killer cell lectin-like receptor (KLR) B1*) were almost exclusively expressed in clusters 7–9, clusters 0–6 and 10–13 displayed a pronounced expression of the myeloid markers *CD68* and *CD14* and the complement *C1QA* chain (Supplementary Fig. 1b). To gain further insights into the different TAM clusters and their type I IFN responses, we re-analysed cells within the myeloid clusters 0–6 and 10–13 separately and clustered them anew. In line with the original analysis^9^, TAM clusters were distinguished into brain-resident microglia (MG-TAMs) (TAM clusters 0, 1, 2, 4, 6, 10, and 12) based on prominent expression of *CX3C motif chemokine receptor 1* (*CX3CR1)*, *excitatory amino acid transporter 1* (*SLC1A3)* and *transmembrane protein 119* (*TMEM119)*, or cells of monocytic origin (MO-TAMs) (TAM clusters 5, 8 and 9), expressing *integrin-α4* (*ITGA4)*, *transforming growth factor-β1* (*TGFBI)* and *formyl peptide receptor 3* (*FPR3)* (Fig. 1a, left panel; Supplementary Fig. 1c). Of note, some cluster populations (3, 7 and 11) did not fit into one of these categories, as they either expressed markers of both subgroups or none of them at all and, thus, could contain dendritic or granulocytic cells. Finally, we used the “hallmark interferon-α response” from MSigDB^29^ to score type I IFN responses between the different TAM clusters. Interestingly, while the highest ISG signature expression was observed in some MG-TAM clusters (2, 10), the clusters containing MO-TAMs (5, 8, 9) rather displayed low to medium ISG signature scores (Fig. 1a, middle and right panel). Of note, there also appeared to be a rather prominent patient-to-patient variability in the analysed samples (Supplementary Fig. 1d). To further investigate type I IFN responses in MO-TAMs in GB, we isolated primary human monocytes from buffy coats and co-cultured them for 48 h with the GB cell line LN229 (Fig. 1b). This experimental setup was chosen to reflect the early interactions between these cell types after monocytes entered the tumour. For downstream analyses of RNA expression changes, monocytes and GB cells were separated by FACS. To identify bona fide IFN targets, we further treated monocytes in the absence of GB with IFN-β (1 ng/ml) for 6 h. Principal component analysis of the expressed genes, determined by bulk RNA-seq analyses, clearly distinguished the three experimental conditions (Supplementary Fig. 1e). It is noteworthy that differentiation along the main principal component (PC1: 66% variance) separated co-culture- and IFN-β-induced mRNA expression profiles from control monocytes in opposite directions. Of the 16,643 genes detected by RNA-seq, 1,076 genes were differentially expressed after IFN-β treatment compared with the control (*padj* < 0.1, |log2FC | > 1). Among these, 317 were down- and 759 were upregulated (Fig. 1c; Supplementary Table 6a), the latter being deemed ISGs. Similarly, we found 929 genes to be differentially expressed between mono- and co-culture (*padj* < 0.1, |log2FC | > 1), 575 being up- and 354 being downregulated (Fig. 1d; Supplementary Table 6b). Of note, annotation of the above-described ISGs revealed a strong enrichment in the cluster with differentially expressed genes (DEGs) downregulated in co-cultured monocytes. In line, while there was a functional enrichment for gene ontology (GO) terms related to immune and inflammatory responses among genes from both clusters, genes downregulated in co-culture appeared to be specifically enriched in antiviral immune responses and type I IFN signalling processes (Fig. 1e; Supplementary Table 7a). Moreover, Gene Set Enrichment Analysis (GSEA) using the Hallmark gene sets from MSigDB not only validated a strong enrichment of the IFN-α response upon treatment with IFN-β but also revealed a depletion of the same set of genes in co-culture (Fig. 1f; Supplementary Table 7b).

These results point towards an attenuated expression of ISGs in primary human monocytes co-cultured with GB cells.

### Type I IFN responses are downregulated in monocytes communicating with glioblastoma cells

**Fig. 2:**
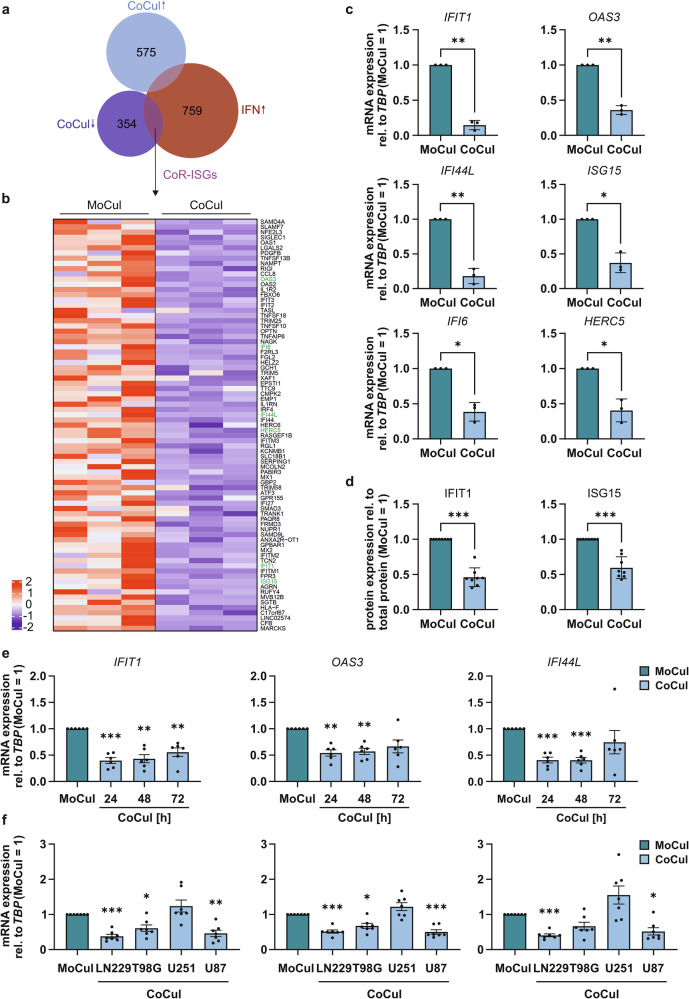
Downregulation of type I IFN responses in monocytes upon contact with glioblastoma cells. **a**, **b**, Primary human monocytes isolated from buffy coats were co-cultured with LN229 cells for 48 h (CoCul) or treated with 1 ng/ml interferon-β (IFN-β) for the last 6 h. RNA expression was determined by RNA-seq. **a**, Venn diagram depicting genes upregulated in monocytes by IFN-β and up- or downregulated after co-culture with LN229 cells (*padj* < 0.1, |log2FC | > 1). **b**, Heatmap visualizing 79 genes (*z*-score normalized counts) upregulated after stimulation with IFN-β and downregulated after co-culture with LN229 cells (co-culture-repressed-ISGs (CoR-ISGs)) from **a**. Selected interferon-stimulated genes (ISGs) are highlighted (*green*). **c**, **d**, Primary human monocytes were mono-cultured (MoCul, *cyan*) or co-cultured (CoCul, *blue*) with LN229 cells in transwell inserts for 48 h. **c**, mRNA expression of selected ISGs (*interferon-induced protein with tetratricopeptide repeats 1* (*IFIT1*), *oligoadenylate synthetase 3* (*OAS3*), *interferon-inducible 44* *L* (*IFI44L*), *interferon-stimulated gene 15* (*ISG15*), *interferon-inducible 6* (*IFI6*), *HECT and RLD domain-containing E3 ubiquitin protein ligase 5* (*HERC5*)) was analysed by qPCR and normalized to *TATA-box-binding protein* (*TBP*) expression (*n* = 3). **d**, Expression of IFIT1 and ISG15 protein was determined by Western blot analysis and normalized to total protein (*n* = 8). **e**, Primary human monocytes were co-cultured with LN229 cells in transwell inserts for the indicated time points (*n* = 6). **f**, Primary human monocytes were co-cultured with various GB cell lines in transwell inserts for 24 h (*n* = 7). **e**, **f**, mRNA expression of *IFIT1*, *OAS3* *and IFI44L* was analysed by qPCR and normalized to *TBP* expression. All data are means ± SEM and were statistically analysed using paired *t* test (**c**, **d**) or one-way repeated measures analysis of variance with Dunnett’s multiple comparisons test (**e**, **f**) (*P < 0.05, **P < 0.01, ***P < 0.001).

To gain further insights into altered ISG expression profiles in monocytes upon co-culture with GB cells, we determined the overlap of the 759 above-described ISGs with the DEGs in co-cultured monocytes (*padj* < 0.1, |log2FC | > 1). While only 9.57% of the 575 upregulated genes in co-culture appeared to be ISGs, a markedly larger proportion of the downregulated genes (i.e. 22.32% of 354) were ISGs (Fig. 2a). The observation that ISGs comprise approximately one quarter of the downregulated genes in co-cultured monocytes, the overlap herein referred to as co-culture-repressed ISGs (CoR-ISGs), indicates that IFN-related signalling might be of major relevance in this context. The CoR-ISGs encompassed many classical ISGs, including *interferon-induced protein with tetratricopeptide repeats* (*IFIT*) *1*, *interferon-inducible* (*IFI*) *6* and *44* *L*, *oligoadenylate synthetase* (*OAS*) *3*, *HECT and RLD domain-containing E3 ubiquitin protein ligase* (*HERC*) *5* and *ISG15* (Fig. 2b; Supplementary Fig. 2a). To further examine if ISG downregulation in monocytes upon co-culture with LN229 cells depends on direct cell-cell contacts, we co-cultured the two cell types in an indirect manner, i.e. using a transwell, Boyden chamber approach (Fig. 2c; Supplementary Fig. 2b). Interestingly, the expression of exemplary ISGs *IFIT1*, *OAS3*, *IFI44L*, *ISG15*, *IFI6* and *HERC5* was also significantly decreased in this indirect co-culture approach compared with monocytes alone, although the reduction was less pronounced than in direct co-culture. In line, IFIT1 and ISG15 protein expression was reduced in monocytes upon 48 h of indirect co-culture with LN229 cells (Fig. 2d; Supplementary Fig. 2c). These findings indicate that soluble factors might contribute to decreased ISG expression in monocytes in the GB context. Importantly, ISG mRNA expression was similarly reduced after 24 and 48 h and was partially lost after 72 h of co-culture (Fig. 2e). As we previously observed differences in ISG expression among different TAM subtypes (Fig. 1a), we wondered if co-culture with LN229 cells might affect ISG expression in microglia as well. Interestingly though, ISG expression was not reduced in C20 or HMC3 microglial cell lines after 24-h co-culture with LN229 cells (Supplementary Fig. 2d). Finally, to determine if the observed ISG downregulation upon co-culture is a LN229-specific phenomenon or if it is a more general response in the GB TME, we co-cultured primary human monocytes with a panel of GB cell lines (LN229, T98G, U87 and U251) for 24 h in the transwell set-up. As co-culture with T98G and U87 cells provoked a similar ISG downregulation in primary human monocytes, this hints towards a broader phenomenon (Fig. 2f). Nevertheless, co-culture with U251 cells did not elicit the same phenotype in monocytes, providing a well-suited negative control for further analyses. Interestingly, a comparison of the basal transcriptomes of the four GB cell lines with GSEA using the C2 curated gene set collection from MSigDB revealed a strong enrichment of the gene set “(Verhaak) glioblastoma multiforme mesenchymal”^3^, which refers to the “mesenchymal” TCGA subtype in LN229, T98G and U87 (REST) compared with U251, whereas the “classical” TCGA subtype was enriched in U251 respective to the other cell lines (Supplementary Fig. 2e; Supplementary Table 7c).

In conclusion, attenuated type I IFN responses upon contact with GB cells appear to be a rather widespread phenomenon in primary human monocytes, but not in microglia, and depend, at least to a major degree, on soluble factors in the TME.

### GM-CSF is a major factor in the glioblastoma-cell-mediated decrease in type I IFN responses in monocytes

**Fig. 3:**
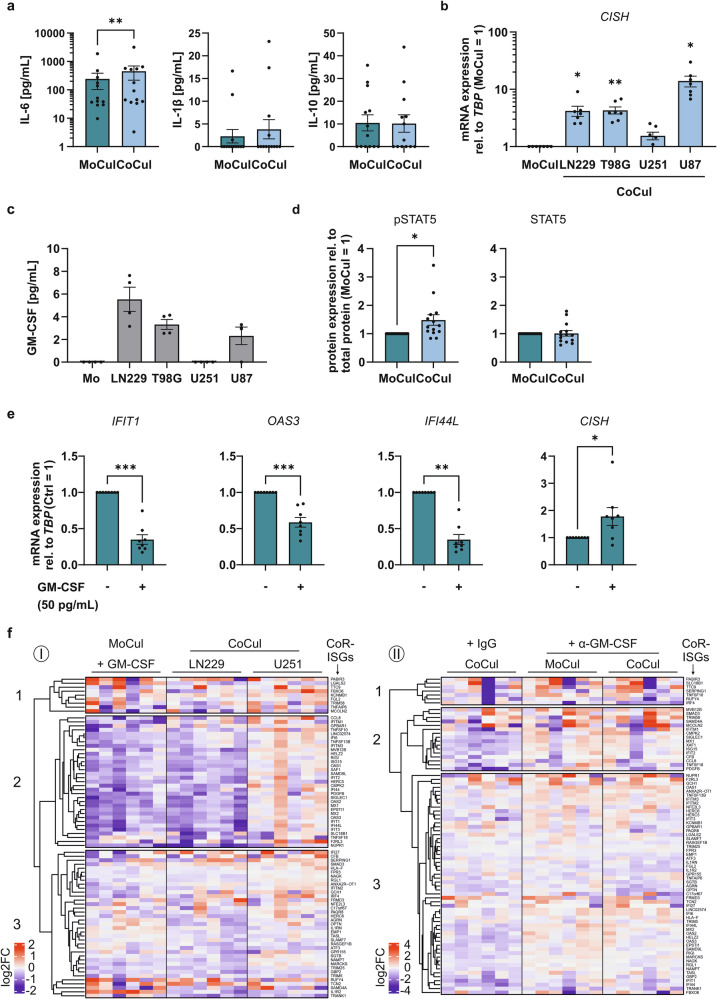
GM-CSF restricts type I IFN responses in monocytes. **a**, Primary human monocytes were mono-cultured (MoCul, *cyan*) or co-cultured (CoCul, *blue*) with LN229 cells in transwell inserts for 24 h. Interleukin (IL)-6, IL-1β and IL-10 protein concentrations in supernatants were determined by cytometric bead array (*n* = 13). **b**, Primary human monocytes were co-cultured with various glioblastoma cell lines in transwell inserts for 24 h (*n* = 7). mRNA expression of the granulocyte-macrophage colony-stimulating factor (GM-CSF) target gene *cytokine-inducible SH2-containing protein* (*CISH*) was analysed by qPCR and normalized to *TATA-box-binding protein* (*TBP*) expression. **c**, GM-CSF protein concentrations in supernatants of several glioblastoma cell lines or primary human monocytes (Mo) were determined by ELISA (*n* = 4). **d**, Primary human monocytes were mono-cultured (MoCul, *cyan*) or co-cultured (CoCul, *blue*) with LN229 cells in transwell inserts for 24 h (*n* = 14). Protein expression of phosphorylated signal transducer and activator of transcription 5 (pSTAT5) (*left panel*) and total STAT5 (*right panel*) was determined by Western blot analysis and normalized to total protein. **e**, Primary human monocytes were treated with recombinant human GM-CSF (50 pg/ml) for 24 h (*n* = 8). mRNA expression of selected interferon-stimulated genes (ISGs) (*interferon-induced protein with tetratricopeptide repeats 1* (*IFIT1*)*, oligoadenylate synthetase 3* (*OAS3*)*, interferon-inducible 44* *L* (*IFI44L*)) and *CISH* was analysed by qPCR and normalized to *TBP* expression. All data are means ± SEM and were statistically analysed using paired *t* test (**a**, **d**, **e**, (*IFIT1*, *OAS3*, *CISH))*, or Wilcoxon matched-pairs signed rank test (**e**, *IFI44L*), or one-way repeated measures analysis of variance with Dunnett’s multiple comparisons test (**b**) (*P < 0.05, **P < 0.01, ***P < 0.001). **f**, Primary human monocytes were co-cultured with LN229 cells or U251 cells in transwell inserts or treated with 50 pg/ml recombinant human GM-CSF for 24 h (I). Monocytes were incubated with 0.5 µg/ml GM-CSF neutralizing antibody (α-GM-CSF), or polyclonal goat IgG control (IgG) and co-cultured with LN229 cells in transwell inserts for 24 h (II). RNA expression was determined by RNA sequencing (*n* = 6). Heatmaps depict log2FC of co-culture-repressed-ISGs (CoR-ISGs) determined in Fig. 2a, categorized by *k*-means clustering.

In order to identify potentially contributing soluble factors, we measured the concentration of classical, TME-associated cytokines (IL-6, IL-1β, IL-10) in supernatants from monocytes and co-cultures of monocytes and LN229 cells (Fig. 3a). In fact, while IL-10 concentrations of both conditions were similar, levels of the pro-inflammatory cytokines IL-6 and IL-1β appeared to be higher in co-culture supernatants. It is noteworthy that both IL-1β and IL-10 levels were generally extremely low (close to or below the detection limit). To assess whether these differences might play a role in reduced ISG expression in monocytes upon co-culture with GB cells, we next evaluated cytokine secretion more broadly (a total of 14 cytokines) in different GB cell lines that had been shown to be differentially effective in regulating ISGs in monocytes (Fig. 2f). As the ISG-repressive properties of the four GB cell lines did not correlate with the concentrations of any of the detectable cytokines (IL-1α, IL-1β, IL-11, IL-12p40, IL-12p70, IL-15, IL-33 or IL6) (Supplementary Fig. 3a and b), they did not appear to be crucial for the observed changes in type I IFN signalling in co-cultures of monocytes and GB cells. Considering that reduced ISG expression upon contact with GB cells was prominent in early monocytes and slightly recovered during their maturation to macrophages (Fig. 2e), we next asked whether factors contributing to the monocyte-to-macrophage maturation process might affect GB-cell-mediated ISG depletion. Therefore, we co-cultured primary human monocytes for 24 h with LN229 cells in the transwell set-up, either with the differentiation factors M-CSF (50 ng/ml), GM-CSF (10 ng/ml), or a combination of the two (50 ng/ml M-CSF plus 10 ng/ml GM-CSF). As observed before, the co-culture-induced ISG repression was prominent in the presence of M-CSF. Strikingly though, GM-CSF by itself (regardless of the presence or absence of additional M-CSF) massively inhibited ISG expression in monocytes (Supplementary Fig. 3c). The notion that co-culture did not further reduce CoR-ISG expression beyond the GM-CSF-induced repression, suggested that the two effects might be related. Therefore, we analysed the expression of the GM-CSF target gene *cytokine-inducible SH2-containing protein* (*CISH*). Interestingly, *CISH* expression was significantly elevated in monocytes co-cultured with different GB cell lines, but not in monocytes co-cultured with U251 (Fig. 3b). In line, GM-CSF was detected in the supernatants of LN229, T98G and U87 cells, but not in the supernatants of U251 cells or monocytes alone (Fig. 3c). Activation of GM-CSF downstream signalling was further confirmed by the upregulation of STAT5 phosphorylation in monocytes upon co-culture with LN229 cells (Fig. 3d; Supplementary Fig. 3d). As the GM-CSF concentrations observed in the supernatants were much lower than those supplemented for differentiation purposes, GM-CSF supplementation was titrated down with respect to its CoR-ISG inhibitory efficacy. Indeed, recombinant human GM-CSF already suppressed ISG expression in primary human monocytes at concentrations of 50 pg/ml to a similar extent to co-cultures with LN229 cells (Fig. 3e; Supplementary Fig. 3f). These results indicate that even very low, pathophysiologically relevant concentrations of GM-CSF might attenuate type I IFN signalling in monocytes in the GB TME. Interestingly, the same concentration of GM-CSF did not reduce CoR-ISG expression in microglial cells (HMC3, C20) (Supplementary Fig. 3e). To gain further insights into the role of GM-CSF in this context, we next determined transcriptome changes of monocytes treated with recombinant human GM-CSF (50 pg/ml), or co-cultured with GM-CSF-producing LN229 versus non-GM-CSF-producing U251 (Fig. 3f-I). In addition, global RNA expression of monocytes co-cultured in the transwell set-up for 24 h with LN229 in the presence or absence of an antibody (0.5 µg/ml) neutralizing GM-CSF (Fig. 3f-II) was analysed. Corroborating the observation in direct co-cultures (Fig. 2b), CoR-ISGs were mostly decreased in indirect co-cultures relative to mono-culture controls (Fig. 3f-l, middle columns; Supplementary Table 6c). Importantly, the same CoR-ISGs were decreased, even to a similar extent, after treatment with human recombinant GM-CSF (Fig. 3f-I, left columns). Specifically, there was a strong decrease in cluster 2 and a less prominent decrease in cluster 3 in both conditions. In contrast, CoR-ISGs were not decreased but rather increased in co-cultures with U251 relative to mono-cultures (Fig. 3f-I, right columns). Co-culture again reduced the expression of CoR-ISGs after IgG-treatment albeit to a lesser extent (Fig. 3f-II, left columns) and neutralization of GM-CSF slightly upregulated CoR-ISG expression in both mono- and co-cultures (Fig. 3f-II, middle and right columns). Rescued CoR-ISG expression due to neutralization of GM-CSF in the co-cultures was further validated via qPCR analyses (Supplementary Fig. 3g).

Taken together, these results suggest that GM-CSF plays a central role in the GB-cell-mediated decrease in ISG expression in monocytes.

### Downregulated IFN-stimulated gene expression in monocytes co-cultured with glioblastoma cells is not dependent on IFNAR

**Fig. 4:**
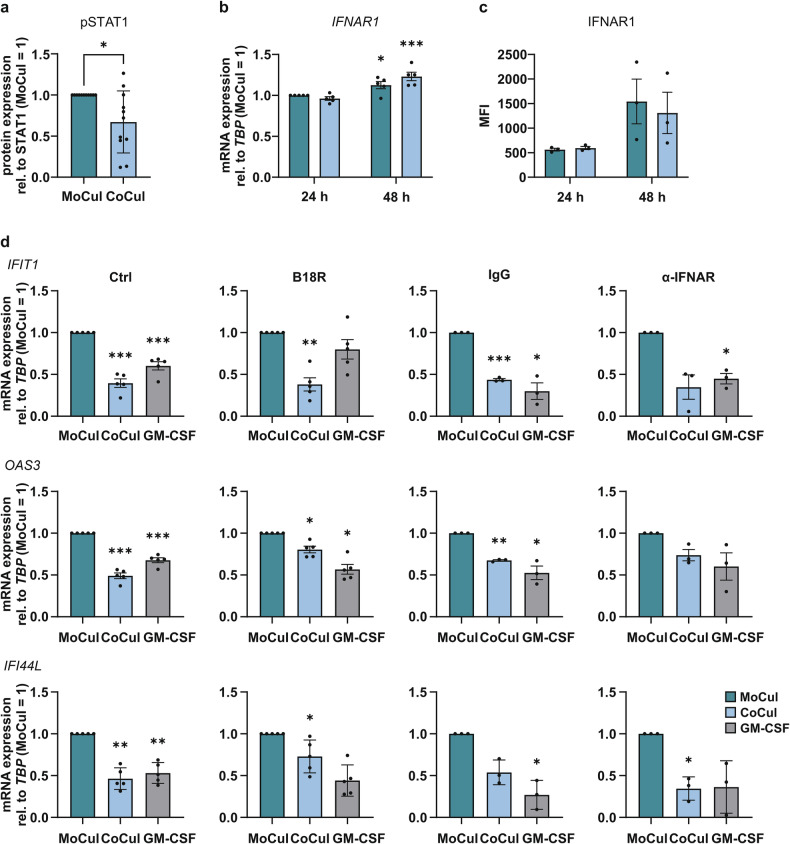
Co-culture-mediated downregulation of type I IFN responses in monocytes is largely independent of IFNAR. **a–c**, Primary human monocytes were mono-cultured (MoCul, *cyan*) or co-cultured (CoCul, *blue*) with LN229 cells in transwell inserts for 24 h (**a**) or for the indicated time points (**b**, **c**). **a**, Phosphorylated signal transducer and activator of transcription 1 (pSTAT1) protein expression was determined by Western blot analysis and normalized to total STAT1 expression (*n* = 11). **b**, *Interferon-α/β-receptor 1* (*IFNAR1*) mRNA expression was analysed by qPCR and normalized to *TATA-box-binding protein* (*TBP*) expression (*n* = 5). **c**, IFNAR1 protein surface expression was analysed using flow cytometry and plotted as median fluorescence intensity (MFI) (*n* = 3). **d**, Primary human monocytes were incubated with 0.1 µg/ml recombinant B18R, or 50 ng/ml IFNAR2 neutralizing antibody (α-IFNAR2) or IgG2a-isotype control (IgG2a), and mono-cultured (MoCul, *cyan*), co-cultured with LN229 cells in transwell inserts (CoCul, *blue*), or treated with 50 pg/ml recombinant human granulocyte-macrophage colony-stimulating factor (GM-CSF, *grey*) for 24 h (*n* ≥ 3). mRNA expression of selected interferon-stimulated genes (ISGs) (*interferon-induced protein with tetratricopeptide repeats 1* (*IFIT1*)*, oligoadenylate synthetase 3* (*OAS3*) and *interferon-inducible 44* *L* (*IFI44L*)) was analysed by qPCR and normalized to *TBP* expression. All data are means ± SEM and were statistically analysed using paired *t* test (**a**), two-way repeated measures analysis of variance (ANOVA) with Šídák’s multiple comparisons test (**b**, **c**) or one-way repeated measures ANOVA with Dunnett’s multiple comparisons test (**d**) (*P < 0.05, **P < 0.01, ***P < 0.001).

As we observed a rather broad attenuation of ISG expression in monocytes co-cultured with GB cells, we next investigated if canonical type I IFN signalling could be involved. We observed a slight decrease in phosphorylation of the major transcription factor STAT1 in co-cultures (Fig. 4a; Supplementary Fig. 4). Therefore, we aimed to investigate the role of IFNAR. While *IFNAR1* expression slightly increased in monocytes between 24 and 48 h after isolation, no differences were detectable between mono- and co-culture in the transwell set-up (Fig. 4b). In line with this, IFNAR1 protein surface expression increased from 24 to 48 h as measured by flow cytometry, again with no differences between mono- and co-culture (Fig. 4c). Of note, IFNAR1 expression after 24 h was only slightly higher than the background (Supplementary Fig. 4b). Therefore, changes in ISG expression in monocytes upon contact with GB cells are not attributable to changes in IFNAR availability. To assess whether IFNAR might still be involved, independent of its availability at the surface, we scavenged type I IFN with the decoy receptor B18R (0.1 µg/ml) or blocked IFNAR2 with a specific antibody (50 ng/ml) in either monocytes co-cultured with LN229 in the transwell set-up or monocytes treated with GM-CSF (50 ng/ml) for 24 h. Both intervention strategies were able to decrease IFN-β-induced upregulation of ISG expression to control levels (Supplementary Fig. 4c). Notably, although both scavenging of type I IFN and neutralization of IFNAR2 strongly reduced basal expression of exemplary ISGs (Supplementary Fig. 4c), GB co-culture- and GM-CSF-mediated downregulation of ISGs was still largely visible after blockade of type I IFN- and IFNAR-mediated signals (Fig. 4d).

Thus, canonical type I IFN signalling via IFNAR was ruled out as playing a major role in the GB co-culture-mediated downregulation of basal type I IFN responses.

### TGF-β contributes to GM-CSF-mediated downregulation of type I IFN signalling in monocytes interacting with glioblastoma cells

**Fig. 5:**
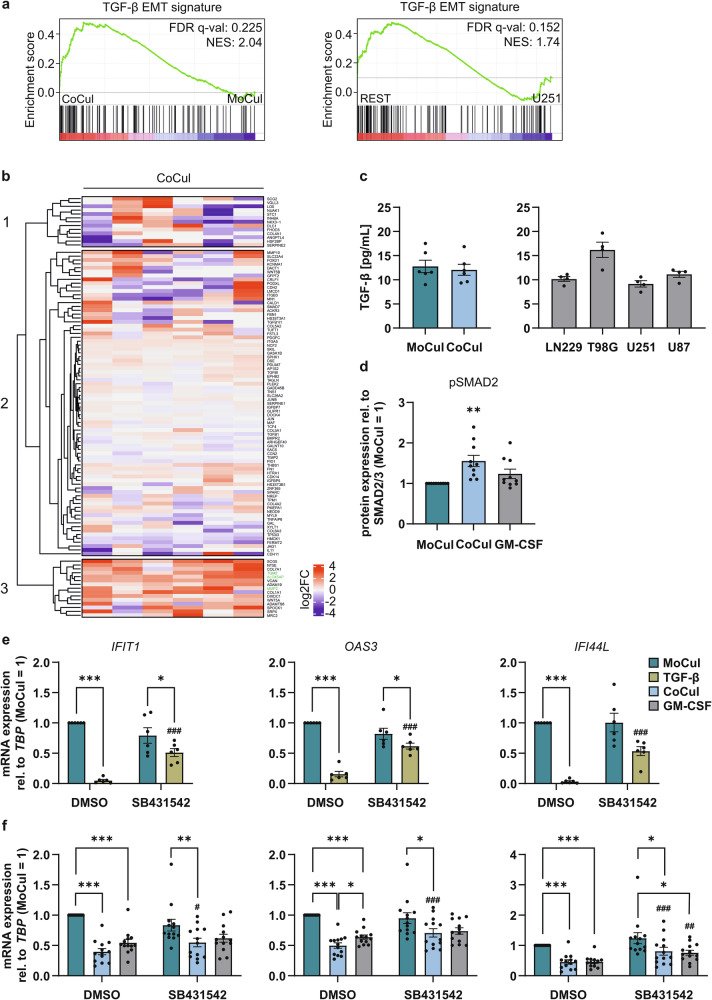
TGF-β contributes to GM-CSF-mediated downregulation of type I IFN signalling in monocytes interacting with glioblastoma cells. **a**, Gene set enrichment analysis of mono-cultured (MoCul) versus co-cultured (CoCul) monocytes, or of LN229, T98G and U87 (REST) versus U251 glioblastoma cells. **b**, Primary human monocytes were co-cultured with LN229 cells in transwell inserts for 24 h. RNA expression was determined by RNA-seq (*n* = 6). Heatmap visualizes log2FC (CoCul relative to MoCul) of genes obtained from gene set “(Foroutan) integrated TGF-β EMT-up”, categorized by *k*-means clustering. **c**, TGF-β protein concentrations in supernatants of mono-cultured (MoCul, *cyan*) or co-cultured (CoCul, *blue*) monocytes (with LN229 cells for 24 h) (left panel) (*n* = 6) or in supernatants of several glioblastoma cell lines (right panel) (*n* = 3) were determined by cytometric bead array. **d**, Primary human monocytes were mono-cultured (MoCul, *cyan*) or co-cultured with LN229 cells in transwell inserts (CoCul, *blue*) or treated with 50 pg/ml recombinant human granulocyte-macrophage colony stimulating factor (GM-CSF, *grey*) for 24 h (*n* = 10). Phosphorylated mothers against decapentaplegic homolog 2 (pSMAD2) protein expression was determined by Western blot analysis and normalized to total SMAD2/3 expression. **e**, Primary human monocytes were treated with 1 ng/ml recombinant human transforming growth factor-β (TGF-β, *yellow*) and an inhibitor for TGF-β signalling (SB431542, 10 µM) for 24 h (*n* = 6). **f**, Primary human monocytes were incubated with SB431542 (10 µM) or DMSO, and mono-cultured (MoCul, *cyan*) or co-cultured (CoCul, *blue*) with LN229 cells in transwell inserts, or treated with 50 pg/ml recombinant human granulocyte-macrophage colony-stimulating factor (GM-CSF, *grey*) for 24 h (*n* = 13). mRNA expression of selected interferon-stimulated genes (ISGs) (*interferon-induced protein with tetratricopeptide repeats 1* (*IFIT1*), *oligoadenylate synthetase 3* (*OAS3*), *interferon-inducible 44* *L* (*IFI44L*)) was analysed by qPCR and normalized to *TATA-box-binding protein* (*TBP*) expression. All data are means ± SEM and were statistically analysed by paired *t* test (**c**, left panel), one-way repeated measures analysis of variance (ANOVA) with Dunnett’s multiple comparisons test (**d**), or two-way repeated measures ANOVA with Šídák’s multiple comparisons test (**e**, **f**) (*P < 0.05, **P < 0.01, ***P < 0.001; ^*#*^*P* < 0.05, ^##^*P* < 0.01, ^*###*^*P* < 0.001 (compared with respective DMSO controls)). FDR, false discovery rate; NES, normalized enrichment score.

To mechanistically understand the attenuation of type I IFN responses in co-cultured monocytes, we examined the RNA-seq data obtained from indirect co-cultures after 24 h (Fig. 3f-I) in more detail using GSEA with the C2 curated gene set collection from MSigDB (Supplementary Table 7d). Herein, “(Foroutan) integrated TGF-β EMT up”^30^ emerged as the top enriched gene set in co-cultured monocytes compared with mono-cultured monocytes (Fig. 5a, left panel). Moreover, the same gene set was enriched in basal transcriptomes of LN229, T98G and U87 (REST) compared with U251 (Fig. 5a, right panel). Visualizing the genes from this gene set expressed in co-cultured monocytes relative to mono-cultured monocytes revealed that a substantial number of TGF-β targets increased in the co-culture, suggesting a robust TGF-β response (Fig. 5b; Supplementary Table 6d). Specifically, while clusters 1 and 2 appeared highly variable, but slightly increased in response to co-culture, cluster 3 contained 15 genes with a strong and consistent upregulation in co-cultures relative to mono-cultures. Importantly, the induction of TGF-β signalling in primary human monocytes co-cultured with GB cells was further substantiated when visualizing the identical signature in the original RNA-sequencing data (Fig. 1) from the direct co-cultures after 48 h (Supplementary Fig. 5a; Supplementary Table 6d). Of note, the clusters with the most upregulated targets (cluster 3) displayed a considerable overlap between direct (Supplementary Fig. 5a) and indirect (Fig. 5b) co-cultures, including genes such as *matrix metalloproteinase 2* (*MMP2*), *transglutaminase 2* (*TGM2*) and *arachidonate 5-lipoxygenase activating protein* (*ALOX5AP*). To investigate whether changes in TGF-β concentration were responsible for the observed signature, we measured TGF-β levels in supernatants from mono- and co-cultures, and in supernatants from different GB cell lines (Fig. 5c). Surprisingly, the presence of TGF-β was similar in mono- and co-cultures and there were also no notable changes among GB cell lines, indicating that other factors derived from GB cells, apart from TGF-β, might have a direct impact on the TGF-β signalling pathway. To find out if GM-CSF could be such a factor, we compared the “(Foroutan) integrated TGF-β EMT up” gene set in mono- and co-cultures treated with either IgG control or with GM-CSF neutralizing antibody (Supplementary Fig. 5b). While the TGF-β signature still appeared to be enriched in co-cultures compared with mono-cultures after IgG treatment, there was no significant enrichment after blockade of GM-CSF, strongly suggesting that GM-CSF is involved in GB-cell-mediated enrichment of TGF-β target genes. In line with this, phosphorylation of mothers against decapentaplegic homolog 2 (SMAD2), i.e. one of the major transcription factors of TGF-β-dependent signalling, was increased in co-cultures, but, to our surprise, barely after treatment with GM-CSF (Fig. 5d; Supplementary Fig. 5c). Stimulation with TGF-β (1 ng/ml) for 24 h and the specific interference with TGF-β signalling using the inhibitor SB431542 (10 µM) confirmed the TGF-β responsiveness of the proposed TGF-β targets (Supplementary Fig. 5d). Interestingly, ISGs also appeared to be highly sensitive to TGF-β, as their expression was strongly reduced after TGF-β stimulation, which was reversed by the inhibition of TGF-β signalling with SB431542 (Fig. 5e). To test whether TGF-β signalling might also contribute to the GM-CSF-mediated attenuation of ISG expression upon co-culture with GB cells, we treated monocytes co-cultured in the transwell set-up with LN229 or stimulated with GM-CSF (50 pg/ml) for 24 h with the TGF-β inhibitor SB431542 (10 µM) (Fig. 5f). Indeed, inhibition of TGF-β signalling slightly increased ISG expression in co-culture, but had only minor effects on ISG expression after GM-CSF treatment. In line with the failure to induce SMAD2 phosphorylation (Fig. 5d), GM-CSF did not induce or only slightly induced TGF-β targets compared with co-culture (Supplementary Fig. 5e). The observation that GM-CSF neutralization still sufficed to suppress the co-culture-induced TGF-β signature (Supplementary Fig. 5b, lower panel), suggests that exogenous recombinant GM-CSF and endogenous GB-cell-derived GM-CSF might differ with respect to their impact on the TGF-β cascade.

Thus, TGF-β signalling appears to be involved in the GM-CSF-mediated downregulation of type I IFN signalling in monocytes during their interaction with GB cells.

### IFN-stimulated gene expression in monocyte-derived tumour-associated macrophages correlates negatively with active GM-CSF signalling in the glioblastoma tumour microenvironment in vivo

**Fig. 6:**
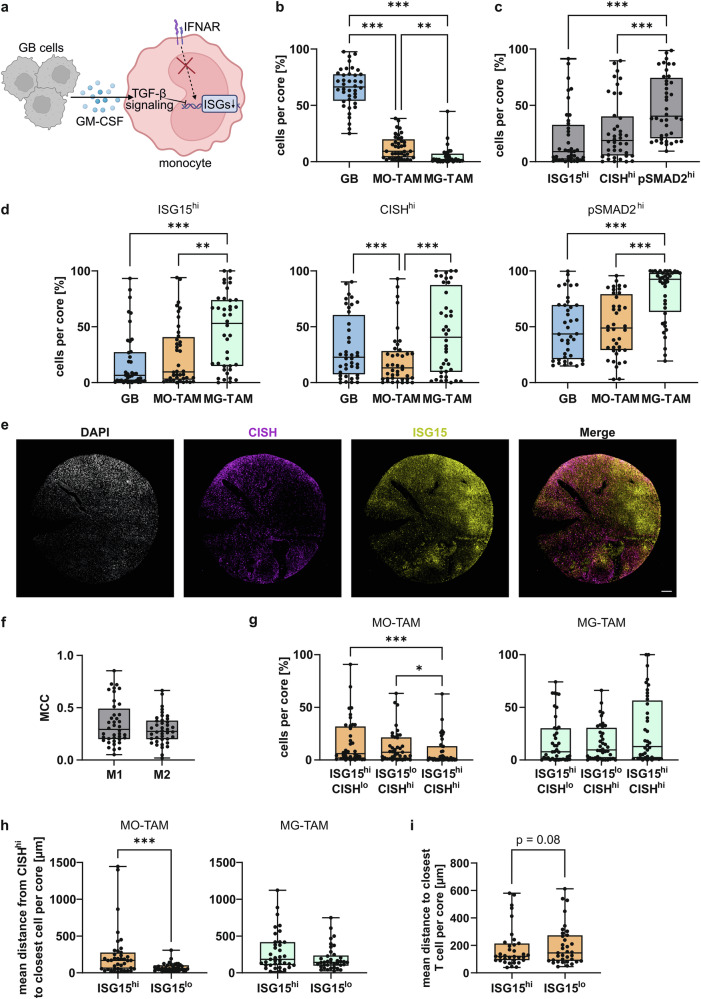
IFN-stimulated gene expression in monocyte-derived tumour-associated macrophages correlates negatively with abundant GM-CSF signalling in the glioblastoma tumour microenvironment in vivo. **a**, Model of the modulation of type I interferon (IFN) responses in monocytes upon contact with granulocyte-macrophage colony-stimulating factor (GM-CSF)-secreting glioblastoma (GB) cells facilitated by transforming growth factor-β (TGF-β) signalling. **b–i**, GB tissue microarrays from 40 patients were analysed by multiplex fluorescent immunohistochemistry using antibodies against glial fibrillary acidic protein (GFAP), ionized calcium-binding adapter molecule 1 (IBA1), transmembrane protein 119 (TMEM119), interferon-stimulated gene 15 (ISG15), cytokine-inducible SH2-containing protein (CISH), phosphorylated mothers against decapentaplegic homolog 2 (pSMAD2) and cluster of differentiation 3 (CD3). **b**, Percentage of tumour cells and residual resident astrocytes (GB; GFAP^+^, *blue*), monocyte-derived tumour-associated macrophages (MO-TAM; IBA1^+^TMEM119^-^, *orange*), and microglial TAMs (MG-TAM; IBA1^+^TMEM119^+^, *green*), or of **c**, ISG15^hi^, CISH^hi^ and pSMAD2^hi^ cells per individual core (*n* = 40). **d**, Percentage of ISG15^hi^, CISH^hi^ and pSMAD2^hi^ cells among tumour cells, MO-TAMs and MG-TAMs. **e**, Representative image of one GB core displaying DAPI (*white*), CISH (*purple*), ISG15 (*yellow*) and the overlay. Scale bar 20 µm. **f**, Manders’ co-localization coefficient (MCC) for each individual core (*n* = 40). M1 depicts fraction of signal from CISH overlapping with ISG15 and M2 depicts fraction of signal from ISG15 overlapping with CISH. **g**, Percentage of MO-TAMs and MG-TAMs with high expression of ISG15, CISH or both. **h**, Mean minimal distance between CISH^hi^ cells and ISG15^hi^ or ISG15^lo^ MO-TAMs or MG-TAMs. **i**, Mean minimal distance between ISG15^hi^ or ISG15^lo^ MO-TAMs and T cells. All data are presented as boxplots depicting median (line), interquartile range (box) and minimum/maximum (whiskers) and were statistically analysed using Friedman test (**b**, **c**, **d**, **g**), or Wilcoxon test (**h**, **i**) (*P < 0.05, **P < 0.01, ***P < 0.001).

Having demonstrated that type I IFN responses are reduced in co-cultured, differentiating monocytes by GB-derived GM-CSF, in part via enhanced TGF-β signalling in vitro (Fig. 6a), we next aimed to validate these findings in tumour biopsies from patients with GB. Therefore, we performed mfIHC to investigate the expression of ISG15 (as a type I IFN target), CISH (as a marker for GM-CSF activity) and nuclear phosphorylated SMAD2 (pSMAD2) (as a proxy for active TGF-β signalling) in GB cells (glial fibrillary acidic protein (GFAP)^+^), MO-TAMs (ionized calcium-binding adapter molecule 1 (IBA1)^+^ transmembrane protein 119 (TMEM119)^−^) and MG-TAMs (IBA1^+^TMEM119^+^) in tissue microarrays from 40 patients with GB (Supplementary Fig. 6a). As expected, GB cells constituted the vast majority of cells within the biopsies (median: 66.35%). Although GFAP is also expressed in resident astrocytes, only a low number of astrocytes are expected in the available sections from tumour cores. Interestingly, MO-TAMs (9.24%) outnumbered MG-TAMs (1.97%) in most biopsies (Fig. 6b), which was even more prominent in GB of the mesenchymal subtype compared with classical and pro-neural subtypes (mesenchymal: 13.29% vs 1.49%, classical/pro-neural: 7.31% vs 2.3%) (Supplementary Fig. 7a). Across all biopsies, 8.94% and 18.84% of the cells were ISG15^hi^ and CISH^hi^, respectively, yet a much larger proportion of all cells displayed signs of activated TGF-β signalling (nuclear p-SMAD2^hi^ 40.41%) (Fig. 6c). Of note, the number of ISG15^hi^ cells was substantially lower in the mesenchymal subtype (4.43%) than in classical and pro-neural subtypes (13.5%), whereas the proportions of cells with high CISH and pSMAD2 expression in the subtypes were rather similar (16% vs 21.8% and 40.41% vs 46.68%, respectively) (Supplementary Fig. 7b). While more than half of the MG-TAMs (53.06%) had high ISG15 expression, most GB cells and MO-TAMs showed low or no expression (6.49% and 9.63%, respectively) (Fig. 6d, left panel), which corroborates the scRNA-seq data, where a higher overall ISG signature expression was observed in MG-TAMs than in MO-TAMs (Fig. 1a). Again, the proportions of cells with high ISG15 expression in all three cell types were especially low in biopsies from the mesenchymal subtype (GB: 3.05%, MO-TAM: 6.84%, MG-TAM: 40.59%) compared with the other subtypes (GB: 13.85%, MO-TAM: 33.07%, MG-TAM: 66.15%) (Supplementary Fig. 7c, left panel). In contrast, markedly more MG-TAMs (40.64%) and GB cells (22.76%) than MO-TAMs (13.14%) expressed high levels of CISH, which was similar across all GB subtypes, hinting at tumour cells and microglia being potential sources of GM-CSF (Fig. 6d, middle panel; Supplementary Fig. 7c, right panel). In line with the overall high percentage of pSMAD2^hi^ cells, the proportions of pSMAD2^hi^ GB cells (43.54%), MO-TAMs (48.89%) and MG-TAMs (92.51%) were substantially higher than those of ISG15^hi^ and CISH^hi^ cells (Fig. 6d, right panel). High pSMAD2 levels further appeared to be present throughout the tissue sections (Supplementary Fig. 6a), suggesting generally elevated TGF-β signalling in GB. Strikingly, ISG15- and CISH-expressing cells localized to distinct, seemingly mutually exclusive regions of the tumour cores (Fig. 6e). Low Manders’ M1 (overlap of CISH signal with ISG15 signal) (0.29) and M2 (overlap of ISG15 signal with CISH signal) (0.27) correlation coefficients (MCCs) confirmed that ISG15- and CISH-expressing cells did not co-localize to a major extent (Fig. 6f). To further validate that ISG15 and CISH were not expressed in the same cells, we determined the proportion of TAMs expressing only one or both markers (Fig. 6g). Interestingly, while a substantially lower proportion of MO-TAMs expressed high levels of both ISG15 and CISH (1.6%) than only one of the markers (6% (ISG15) or 7.36% (CISH)), cells expressing single or both markers at high levels were rather evenly distributed in MG-TAMs (7.76% (ISG15), 9.61% (CISH) or 12.8% (both)). In line with previous observations, the proportion of ISG15^hi^ tumour cells was low, regardless of co-expression with CISH (Supplementary Fig. 6b). To gain further insights into the impact of GM-CSF on type I IFN responses in GB specimens, we determined the minimum distances between each CISH^hi^ cell and the next ISG15^hi^ or ISG15^lo^ cell for the different cell types. In line with the in vitro observations of monocytic versus microglial cells, the mean minimum distance between CISH^hi^ cells and the next MG-TAM appeared to be independent of the ISG15 expression of the latter (Fig. 6h, right panel), whereas ISG15^lo^ MO-TAMs and tumour cells were located significantly closer to CISH^hi^ cells compared with ISG15^hi^ cells (Fig. 6h, left panel; Supplementary Fig. 6c). Finally, to assess if the GM-CSF-dependent decrease in ISG expression specifically in MO-TAMs might have functional consequences for the GB TME, we stained the tissue microarray for the T cell marker CD3 to determine T cell recruitment (Supplementary Fig. 6d). As expected, T cell abundance was very low across all tumour samples (0.54%) (Supplementary Fig. 6e). Interestingly, the proportion of ISG15^lo^ MO-TAMs correlated negatively with the proportion of T cells (Supplementary Fig. 6f), suggesting that T cell infiltration into GB partly depends on type I IFN responses specifically in MO-TAMs. We further analysed the minimum distance between each ISG15^hi^ or ISG15^lo^ MO-TAM and the next T cell and found that T cells were located substantially closer to MO-TAMs with high ISG15 than with low ISG15 expression (Fig. 6i).

Taken together, in line with the in vitro observations, ISG expression in MO-TAMs in GB in situ appears to be reduced by GM-CSF, likely produced by tumour cells or MG-TAMs. Specifically, MO-TAMs with low ISG expression due to GM-CSF signals in close proximity have a reduced capacity to recruit T cells, thereby inhibiting anti-tumour T cell responses in GB.

## Discussion

In the present study, we show that type I IFN responses are inhibited in monocyte-derived cells within the GB TME owing to tumour-cell-derived signals in their immediate vicinity. Specifically, we observed that co-cultivation of primary human monocytes with GB cells downregulated type I IFN responses. While this decrease in ISG expression was largely independent of the classical type I IFN receptor IFNAR, we identified GB-cell-secreted GM-CSF as a critical mediator attenuating monocytic ISG expression. We further provide evidence of a supporting role of TGF-β in the GM-CSF-induced repression of type I IFN responses. Finally, we translate these findings to primary GB tissue, where GM-CSF and type I IFN responses appeared to be mutually exclusive, and ISG-expressing MO-TAMs were absent in the direct vicinity of GM-CSF-producing cells. Functionally, low ISG expression in MO-TAMs correlated with a reduced recruitment of T cells, and thus anti-tumour immune activities.

Type I IFNs usually convey immunostimulatory and tumour-suppressive effects in the TME, especially when they act on myeloid cells^20,31^. In GB, type I IFN signalling in monocytes and their descendants appears to be of crucial importance, as they are often both primary source and target of type I IFNs^32^. We observed that primary human monocytes co-cultured with different GB cell lines displayed reduced expression of ISGs in vitro. This reveals GB-specific features, as contrary findings have been made in other tumour models, for example, lung cancer and melanoma, where in vitro culture of macrophages together with tumour cells or in tumour-conditioned medium increased the expression of selected ISGs^33,34^. In contrast to our findings, Klemm and colleagues demonstrated not only increased ISG expression in macrophages cultured in GB-conditioned medium but also an enriched type I IFN signature in MO- and MG-TAMs from glioma compared with microglia from healthy brain or in vitro-generated MO-TAMs^8^. Similarly, murine TAMs isolated from GL261-derived GB displayed an enriched type I IFN signature in comparison with naïve microglia or peritoneal macrophages^35^. These differences might result from the high tumour cell heterogeneity, which is a hallmark of GB, and the different GB subtypes, which have been described based on genetic, transcriptomic and epigenetic profiles^3,36,37^. Indeed, we found reduced type I IFN responses in monocytes in co-cultures with three GB cell lines, i.e. LN229, T98G and U87 but not with U251, which, in contrast, rather increased ISG expression. Interestingly, while the basal U251 transcriptome reflects the “classical” TCGA subtype, the other three GB cell lines share a transcriptomic profile similar to the “mesenchymal” TCGA subtype, which is also the subtype with the highest inflammatory signature and the highest TAM content^38^. The higher ISG-repressive capacity of GBs of a mesenchymal subtype compared with others was also in line with our in vivo data indicating a substantially lower number of MO-TAMs, with high ISG expression in GB specimen with the mesenchymal compared with other subtypes. Moreover, tumour-associated myeloid-cell populations are equally heterogeneous; for example, different monocyte subsets with different ISG profiles exist in different cancers^39^. Our finding that co-culture-mediated reduction of type I IFN responses was specific to primary monocytes but not microglia corroborates re-analysis of published data from co-cultures of microglial cell lines with GB cells for ISG expression (data not shown^28^). Importantly, although TAMs are commonly considered to be immunosuppressive, and even specifically so in monocytes co-cultured with GB cells^40^, our data rather suggest a broad pro-inflammatory transcriptomic and secreted cytokine profile, indicating that the depletion of a type I IFN signature was a specific trait. While in the latter example, direct cell–cell contact with GB cells was necessary to provoke immunosuppressive properties in monocytes, we found that type I IFN responses also decrease, although to a lesser extent, in an indirect co-culture model, allowing only for the exchange of soluble factors. We identified GM-CSF as a critical factor secreted by GB cells, which contributes to reduced ISG expression in monocytes. GM-CSF is an important growth factor for myeloid-cell generation, function and survival^41^. Moreover, GM-CSF was shown to provoke an inflammatory profile in monocytes and is commonly used to generate pro-inflammatory (previously termed M1-) macrophages^42^. Nevertheless, we observed that GM-CSF elicited a strong inhibitory effect on type I IFN response gene expression, which corroborates earlier reports showing higher sensitivity to vesicular stomatitis virus infection, lower expression and secretion of type I IFN and selected ISGs, as well as decreased interferon-regulatory factor 3 (IRF3) and STAT1 phosphorylation in response to IFN-α or LPS in GM-CSF- compared with M-CSF-polarized macrophages^43–45^. However, our study did not target the role of differentiation or polarization in type I IFN responses; instead we focused on the immediate effect of GM-CSF on ISG expression in monocytes. Similarly, Kasper and colleagues found reduced STAT1, STAT2, JAK1 and tyrosine kinase 2 (TYK2) phosphorylation and expression of selected ISGs in monocytic cell lines after 24 and 48 h incubation with GM-CSF^46^. Our findings go markedly beyond these reports, as we observed a global decrease in type I IFN responses already in response to extremely low concentrations of GM-CSF (low pg/ml). In addition, we proved the link between GM-CSF and reduced type I IFN responses in the co-culture with GB cells via GM-CSF neutralization experiments.

Our data further indicate that TGF-β signalling is involved in the downregulation of ISGs in monocytes upon contact with GB cells. TGF-β is an immunosuppressive cytokine allowing for immune evasion of tumours by interfering with T cell proliferation and effector functions, natural killer cell killing capacity and antigen presentation^47^. Whereas the immunomodulatory functions of TGF-β and its role in the immunosuppressive polarization of macrophages are well characterized^48,49^, there are only a few reports on a type I IFN-inhibitory function. For example, TGF-β was shown to decrease type I IFN signalling in mouse alveolar macrophages and in spontaneous murine mammary tumours^50,51^. In line with this, we found downregulated ISG expression in primary human monocytes stimulated with TGF-β. Interestingly, however, although we observed a strong enrichment of a TGF-β EMT signature in monocytes co-cultured with LN229 and in the basal transcriptomes of GM-CSF-expressing GB cell lines (LN229, T98G and U87) compared with U251, this enrichment was not attributable to changes in TGF-β secretion. Our data rather suggest that GM-CSF directly activates TGF-β signalling independent of TGF-β protein, as neutralization of GM-CSF overcame expression of the TGF-β signature. Surprisingly though, exogeneous recombinant GM-CSF had only a minor effect on TGF-β signalling and target gene expression. Moreover, downregulation of ISG expression in co-cultures was overcome by inhibition of TGF-β signalling, whereas GM-CSF-dependent ISG repression was only minimally rescued. This discrepancy might be due to differences in the biological activity of exogenously supplied and endogenously produced GB-cell-derived GM-CSF.

We identified GM-CSF and TGF-β as early mediators during the interaction of monocytes and GB cells, which set the stage for downstream regulation that ultimately results in reduced expression of ISGs and consequently interferes with type I IFN signalling, notably, via reduced phosphorylation of STAT1, but without major involvement of the central type I IFN receptor IFNAR. As some of the most prominent type I IFN signalling inhibitory mediators were shown not to be involved in GM-CSF-mediated ISG downregulation so far, including the suppressors of cytokine signalling (SOCS) protein family and the protein-tyrosine phosphatases SHP1 and SHP2 (ref. 46), it remains to be determined which mechanisms dictate reduced type I IFN signalling in response to GM-CSF and TGF-β.

Furthermore, we confirmed decreased type I IFN responses in MO-TAMs in the GB TME under the influence of GM-CSF in vivo. Although our approach did not allow for a distinction between GB-associated monocytes and naïve monocytes, we were able to compare ISG expression in MO-TAMs and MG-TAMs. Both published scRNA-sequencing data and multispectral imaging analysis of GB tissue microarrays revealed a lower type I IFN target signature expression in MO-TAMs than in MG-TAMs. As a side note, a higher type I IFN signature was previously shown in GB-associated microglia relative to homeostatic microglia^52^. Moreover, type I IFN responses and the GM-CSF target CISH were spatially separated, and the neighbourhood of CISH^hi^ cells harboured only a few MO-TAMs with high ISG expression, but MG-TAMs with high and low ISG expression alike. Thus, our in vitro observation of reduced ISG expression by GB-cell-derived GM-CSF in monocytes, but not microglia, appears to be specific to MO-TAMs in the GB TME. We further differentiated between specimens that were attributed to the mesenchymal or other subtypes and found an overall very low ISG expression, most prominently in MO-TAMs in mesenchymal GB, resembling our in vitro finding that type I IFN responses are specifically inhibited in monocytes upon contact with GB cell lines reflecting the mesenchymal subtype. As GBs are commonly composed of cells from different subtypes, one of which usually dominates, it will be interesting to analyse the effect of single tumour cells assigned to various subtypes on neighbouring TAMs in more detail in future studies. Interestingly, high levels of GM-CSF were measured in tumour specimens and serum of GB patients and put forward as predictors of a poor prognosis^53,54^. Our findings contribute to the larger picture of the dichotomous properties of GM-CSF, which was originally considered as a pro-inflammatory stimulus and anti-tumoural agent, but turned out to have highly context-dependent immunosuppressive and pro-tumourigenic effects^55^. Of note, the high TGF-β activity found in the GB specimen corroborates previous reports showing that high TGF-β levels are found in many tumour entities and that elevated TGF-β expression was specifically abundant in GB specimens compared with normal brain tissue^56,57^. Nevertheless, the homogenous distribution of active TGF-β signals across the GB tissue microarrays prevented the in vivo validation of the important role of TGF-β during GB-cell-mediated downregulation of type I IFN responses in monocytes in vitro. Future studies are needed to decipher the differences in intracellular TGF-β signalling in GB-TAMs in vivo and the potential impact on type I IFN signalling.

Our data further provide first indications of the functional consequences of decreased type I IFN responses in MO-TAMs in the GB TME. As we observed a negative correlation of T cell abundance with the amount of ISG-repressive MO-TAMs and a closer proximity of T cells to MO-TAMs with high ISG expression, we propose that type I IFN responses in MO-TAMs affect their capacity to recruit T cells. Similar connections between T cell infiltration into tumours and type I IFN signalling in tumour-associated myeloid cells have been described before^58–60^. Notably, one of the most important T cell chemoattractants, CXCL10, is an interferon-regulated cytokine^61^. Thus, given the pro-inflammatory and tumour-suppressive properties of type I IFNs and of monocytes with an enriched ISG signature^20^, we hypothesize that IFN-depleted MO-TAMs contribute to the immunosuppressive modulation of the GB TME, and consequently to the devastating prognosis of this disease, which makes further research on type I IFN responses in TAMs in GB essential. It is noteworthy that we previously found that hypoxia boosts the expression of ISGs in monocytes in an infection context^61^. It will thus be interesting to see how hypoxia, which is a typical feature of the GB TME, might affect ISG expression in the crosstalk of monocytes and GB cells.

Taken together, we characterize a type I IFN-inhibitory pathway during the early interaction of tumour cells and peripheral monocytes in the GB TME. The identification of GM-CSF and TGF-β as major factors may pave the way for novel therapeutic strategies.

## Supplementary information

Supplementary Information

Supplementary Data1

Supplementary Data2

## Data Availability

Sequencing data are available in GEO (accession numbers GSE309037, GSE309038, GSE309039). Supporting data are available in the Supplementary Information.
