# Supplementary material for: GM-CSF downregulates type I IFN responses in glioblastoma-associated monocytes
Source: Exp Mol Med. 2026 Jul 3;58(7):2252–67. doi: 10.1038/s12276-026-01769-1 (PMC13434229; doi:10.1038/s12276-026-01769-1)
Supplement: Supplementary file 1 [file 12276_2026_1769_MOESM1_ESM.pdf]

<u>**SUPPLEMENTARY INFORMATION**</u>

Supplementary Figures

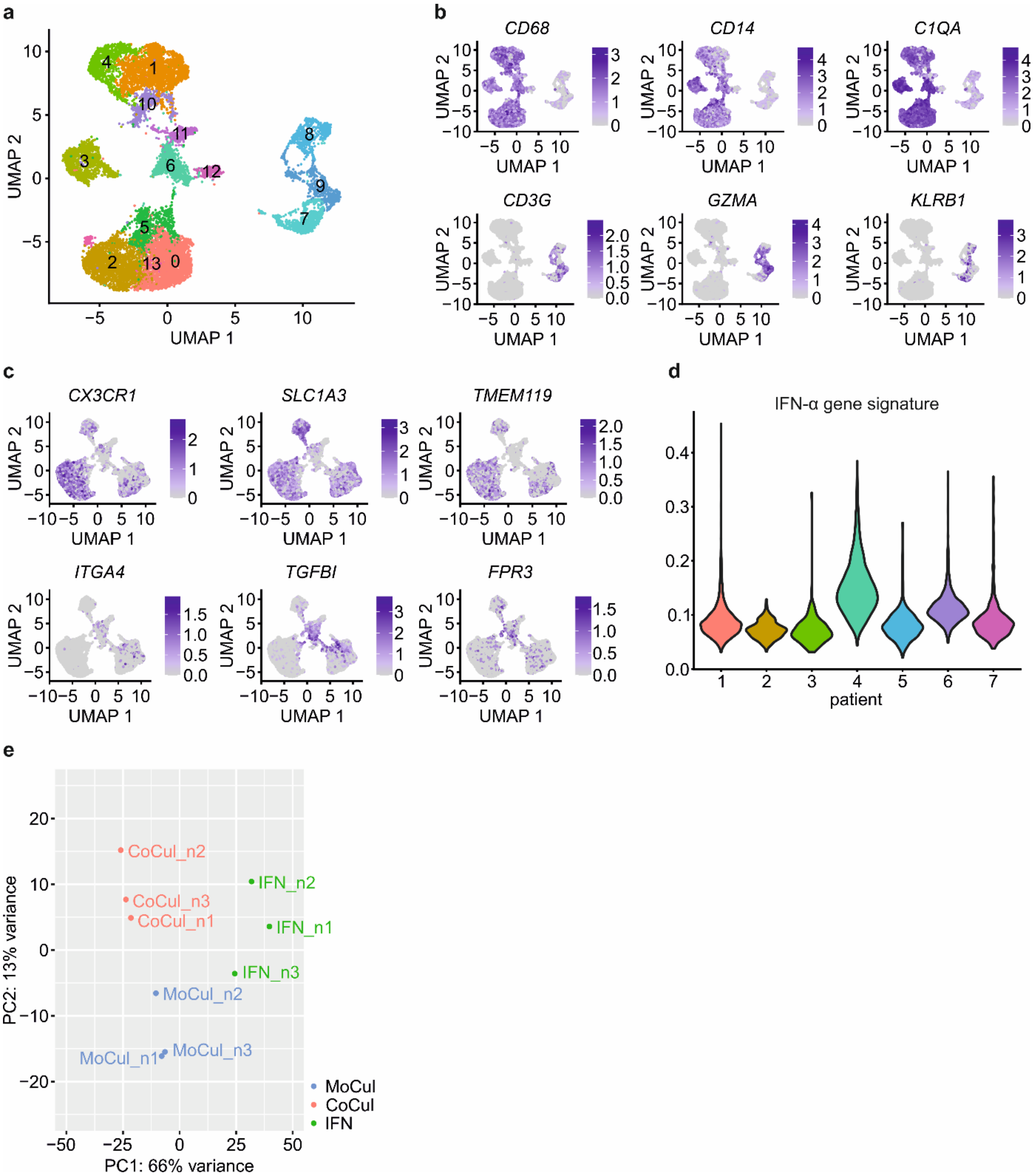

Supplementary Fig. 1: Differential type I IFN-stimulated gene (ISG) expression in tumor-associated monocytes and macrophages in the GB tumor microenvironment. (**a**) Uniform manifold approximation and projection (UMAP) of CD45-positive cells from seven patients with newly diagnosed GBM[1]. Feature plot visualization of the relative expression of genes characteristic for different (**b**) immune cell types (*CD68*: macrophages; *CD14*: monocytes; complement *C1qA* chain (*C1QA*): macrophages; *CD3G*: T cells; *granzyme A* (*GZMA*): cytotoxic cells; *killer cell lectin-like receptor B1 (KLRB1)*: NK cells) or (**c**) specific tumor-associated macrophage (TAM) subtypes (monocyte-derived: *integrin-α4* (*ITGA4*),

transforming growth factor-β1 (*TGFBI*), *formyl peptide receptor 3* (*FPR3*); microglial: *CX3C motif chemokine receptor 1* (*CXCR1*), *excitatory amino acid transporter 1* (*SLC1A3*), *transmembrane protein 119* (*TMEM119*)). (**d**) Violin plots of an interferon (IFN)-α gene signature scored with UCell in CD45-positive cells with substantial expression of myeloid markers across different patients. (**e**) Primary human monocytes were isolated from buffy coats using CD14-MACS, mono-cultured (MoCul) or co-cultured (CoCul) with LN229 cells for 48 h or treated with 1 ng/mL interferon-β (IFN) for the last 6 h. Monocytes were purified from co-cultures by fluorescence-activated cell sorting (FACS). RNA expression was determined by RNA-seq (n = 3). Graph depicts principal component analysis of normalized read counts of all genes.

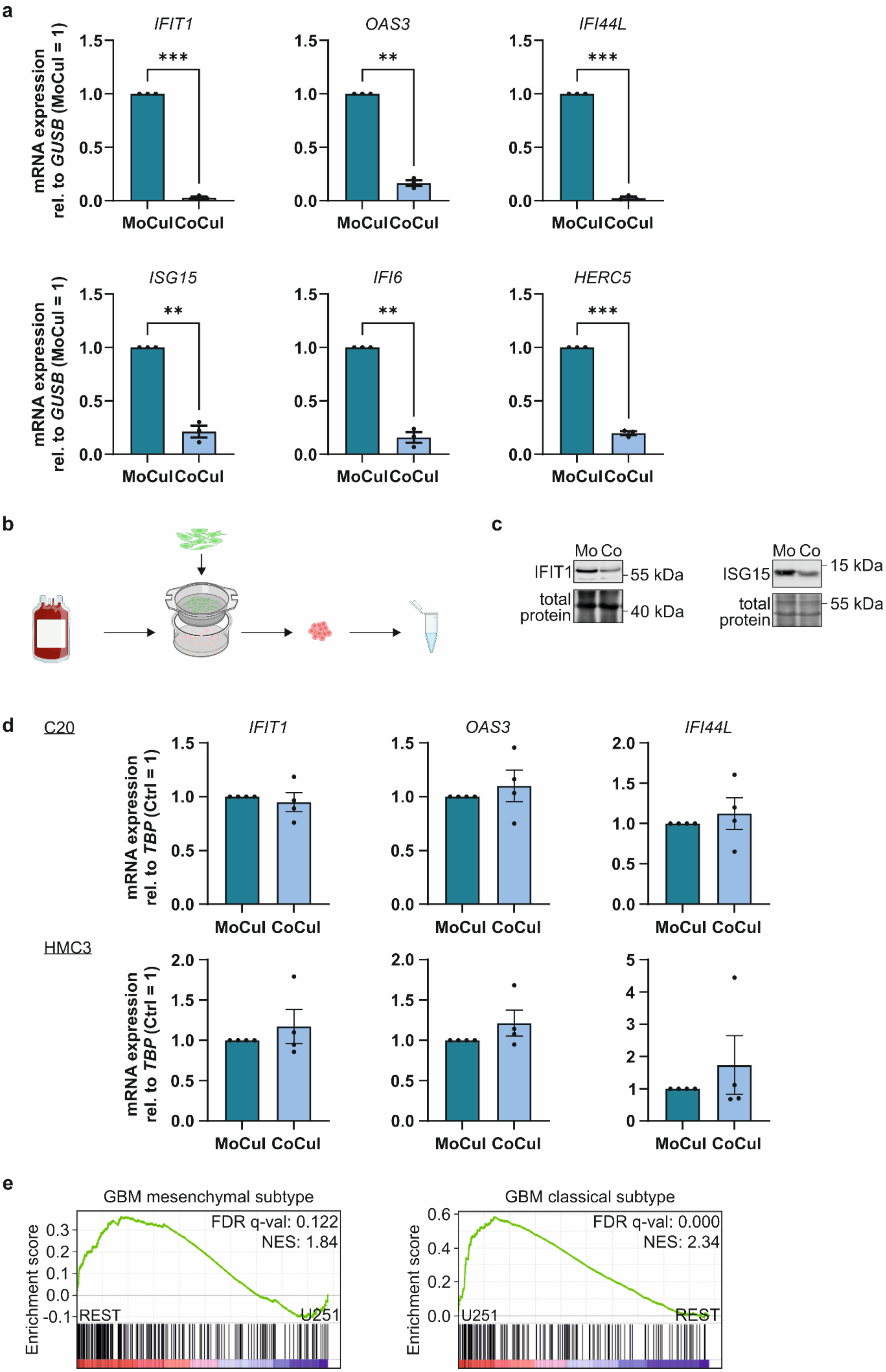
a
IFIT1
OAS3
IFI44L
ISG15
IFI6
HERC5
mRNA expression rel. to GUSB (MoCul = 1)
***
**
1.5
1.0
0.5
0.0
MoCul CoCul
b
c
Mo Co
IFIT1
55 kDa
total protein
40 kDa
ISG15
15 kDa
55 kDa
d
C20
HMC3
mRNA expression rel. to TBP (Ctrl = 1)
2.0
5
4
3
2
1
0
e
GBM mesenchymal subtype
FDR q-val: 0.122
NES: 1.84
GBM classical subtype
FDR q-val: 0.000
NES: 2.34
Enrichment score
0.3
0.2
0.1
0.0
-0.1
0.6
0.4
REST
U251

Supplementary Fig. 2: Type I IFN responses are downregulated in monocytes communicating with GB cells. (**a**) Primary human monocytes were mono-cultured (MoCul, *cyan*) or co-cultured (CoCul, *blue*) with LN229 cells for 48 h (n = 3). mRNA expression of selected interferon-stimulated genes (ISGs) (*interferon-induced protein with tetratricopeptide repeats 1* (*IFIT1*)*, oligoadenylate synthetase 3* (*OAS3*)*, interferon-inducible 44L* (*IFI44L*)*, interferon-stimulated gene 15* (*ISG15*)*, interferon-inducible 6* (*IFI6*)*, HECT and RLD domain-containing E3 ubiquitin protein ligase 5* (*HERC5*)) was analyzed by qPCR and normalized to *glucuronidase β* (*GUSB*) expression. (**b**) Primary human monocytes were mono-cultured or co-cultured with LN229 cells in transwell inserts for 48 h (experimental procedure) (Figure created in BioRender. Schmid, T. (2026) https://BioRender.com/dmj8o9g). (**c**) IFIT1 (*left panel*) and ISG15 (*right panel*) protein expression was determined by Western blot analysis and normalized to total protein. Blots are representative of eight independent experiments. (**d**) C20 (*upper panel*) or HMC3 (*lower panel*) human microglial cells were mono-cultured (MoCul, *cyan*) or co-cultured (CoCul, *blue*) with LN229 cells for 24 h (n = 4). mRNA expression of *IFIT1, OAS3,* or *IFI44L* was analyzed by qPCR and normalized to *TATA-box-binding protein* (*TBP*) expression. All data are means ± SEM and were statistically analyzed by paired t-test (**p* < 0.05, ***p* < 0.01, ****p* < 0.001). (**e**) Gene Set Enrichment Analysis (GSEA) of untreated LN229, T98G, and U87 cells (REST) vs. U251 cells (*left panel*), or U251 cells vs. REST (*right panel*) (FDR: false discovery rate; NES: normalized enrichment score).

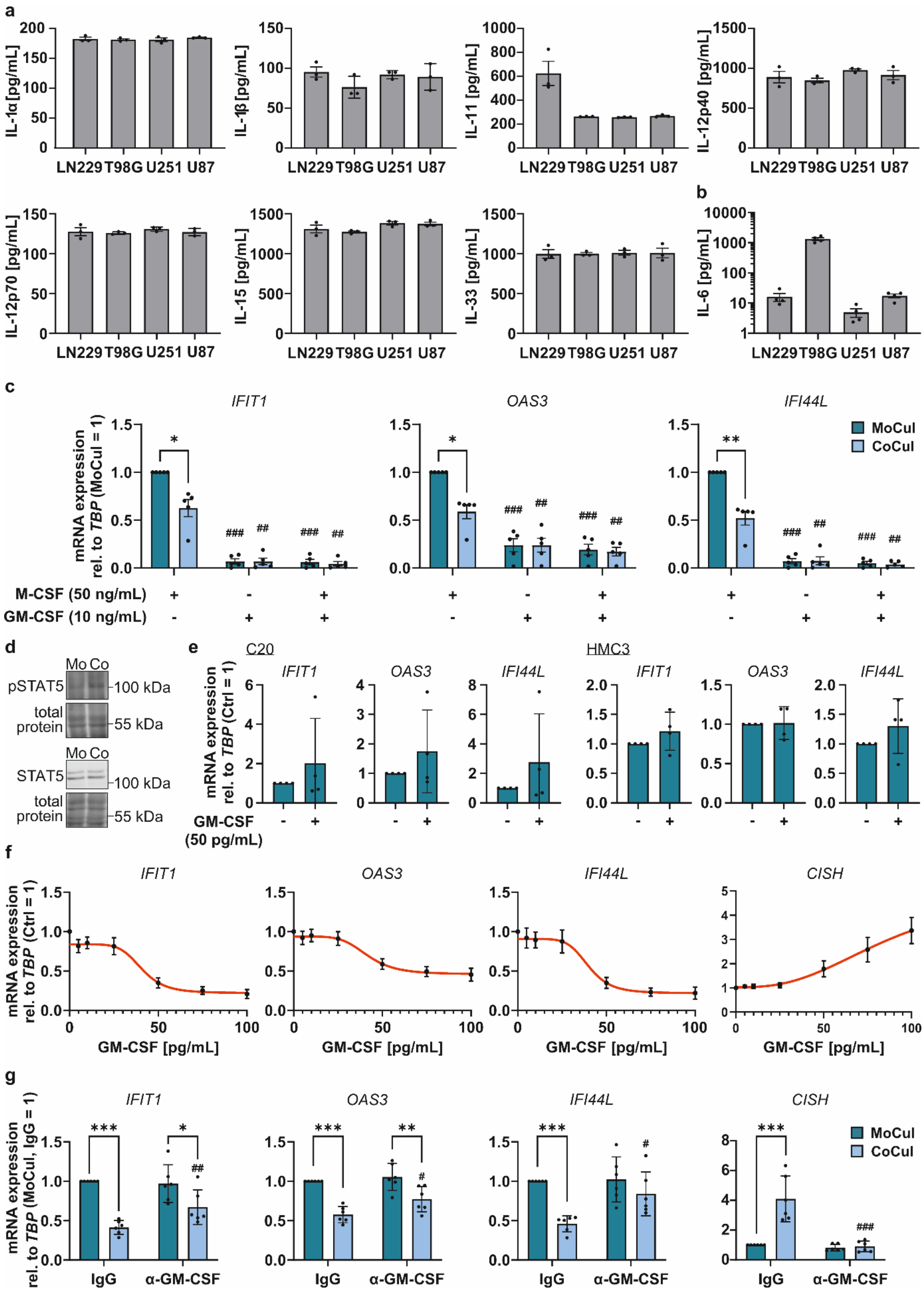

Supplementary Fig. 3: GM-CSF is a major factor in the GB cell-mediated decrease in type I IFN responses in monocytes. (**a, b**) Interleukin (IL)-1α, IL-1β, IL-11, IL-12p40, IL-12p70, IL-15, IL-33, and IL- 6 protein concentrations in supernatants of several GB cell lines were determined by LegendPlex (**a**) (n = 3) or cytometric bead array (**b**) (n = 4). (**c**) Primary

human monocytes were mono-cultured (MoCul, *cyan*) or co-cultured (CoCul, *blue*) with LN229 cells in transwell inserts in medium containing either recombinant human macrophage-colony stimulating factor (M-CSF) (50 ng/mL), granulocyte-macrophage-colony stimulating factor (GM-CSF) (10 ng/mL), or the combination of both for 24 h (n = 5). mRNA expression of selected interferon-stimulated genes (ISGs) (*interferon-induced protein with tetratricopeptide repeats 1* (*IFIT1*), *oligoadenylate synthetase 3* (*OAS3*), *interferon-inducible 44L* (*IFI44L*)) was analyzed by qPCR and normalized to *TATA-box-binding protein* (*TBP*) expression. (**d**) Phosphorylated signal transducer and activator of transcription 5 (pSTAT5) (*upper panel*) and total STAT5 (*lower panel*) protein expression was determined by Western blot analysis and normalized to total protein. Blots are representative of 14 independent experiments. (**e**) C20 (*left panel*) or HMC3 (*right panel*) human microglial cells were treated with recombinant human GM-CSF (50 pg/mL) for 24 h (n = 4). (**f**) Primary human monocytes were treated with different concentrations of recombinant human GM-CSF for 24 h (n = 6). (**g**) Monocytes were incubated with 0.5 µg/mL GM-CSF neutralizing antibody (α-GM-CSF) or polyclonal goat IgG control (IgG) and mono-cultured (MoCul, *cyan*) or co-cultured (CoCul, *blue*) with LN229 cells in transwell inserts for 24 h (n = 6). mRNA expression of *IFIT1*, *OAS3*, *IFI44L*, and the GM-CSF target gene *cytokine-inducible SH2-containing protein* (*CISH*) was analyzed by qPCR and normalized to *TBP* expression. All data are means ± SEM. Sigmoidal 4-parameter logistic curve interpolation was performed with GraphPad Prism (**f**), and data were statistically analyzed by two-way repeated measures ANOVA with Šídák's multiple comparisons test (**c, g**) or paired t-test (**e**) ($^{*}p < 0.05$, $^{**}p < 0.01$, $^{***}p < 0.001$; $^{\#}p < 0.05$, $^{\#\#}p < 0.01$, $^{\#\#\#}p < 0.001$ (compared to respective M-CSF controls (**c**) or respective IgG controls (**g**))).

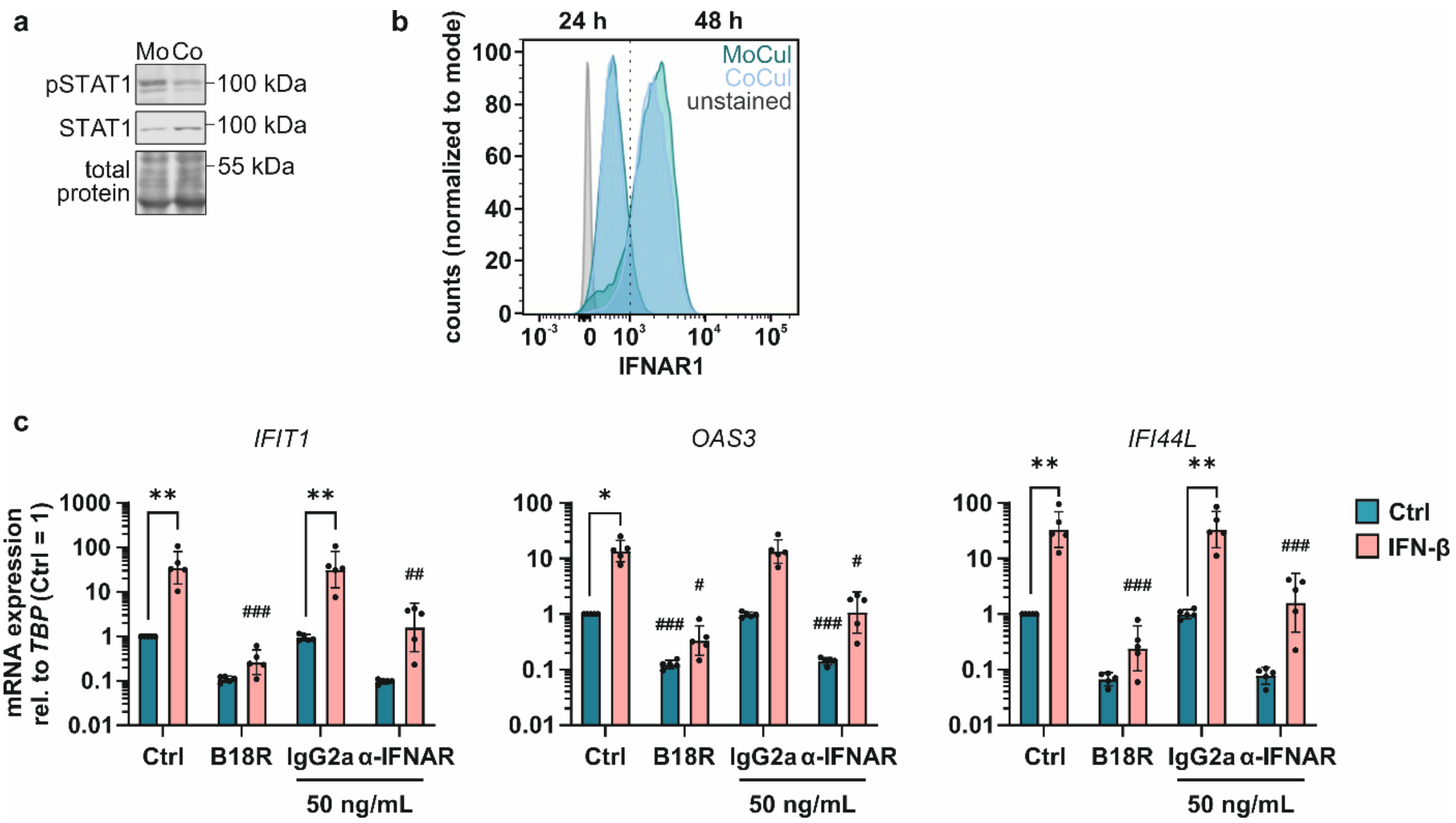

Supplementary Fig. 4: Co-culture-mediated downregulation of type I IFN responses in monocytes is independent of IFNAR. (**a**) Phosphorylated signal transducer and activator of transcription 1 (pSTAT1) protein expression was determined by Western blot analysis and normalized to total STAT1. Blots are representative of 11 independent experiments. (**b**) IFNAR1 protein surface expression was analyzed using flow cytometry. The histogram is representative of three independent experiments. (**c**) Primary human monocytes were incubated with 0.1 µg/mL recombinant B18R, or 50 ng/mL interferon-α/β-receptor 2 (IFNAR2) neutralizing antibody (α-IFNAR) or IgG2a-isotype control (IgG2a) for 24 h and treated with 1 ng/mL interferon (IFN)-β for the last 6 h (n = 5). mRNA expression of selected interferon-stimulated genes (ISGs) (*interferon-induced protein with tetratricopeptide repeats 1* (*IFIT1*), *oligoadenylate synthetase 3* (*OAS3*), *interferon-inducible 44L* (*IFI44L*)) was analyzed by qPCR and normalized to *TATA-box-binding protein (TBP*) expression. All data are means ± SEM and were statistically analyzed by mixed-effects analysis with Šídák's or Tukey's multiple comparisons test (*$p < 0.05$, **$p < 0.01$, ***$p < 0.001$, #$p < 0.05$, ##$p < 0.01$, ###$p < 0.001$ (compared to respective controls (Ctrl for B18R, IgG2a for α-IFNAR))).

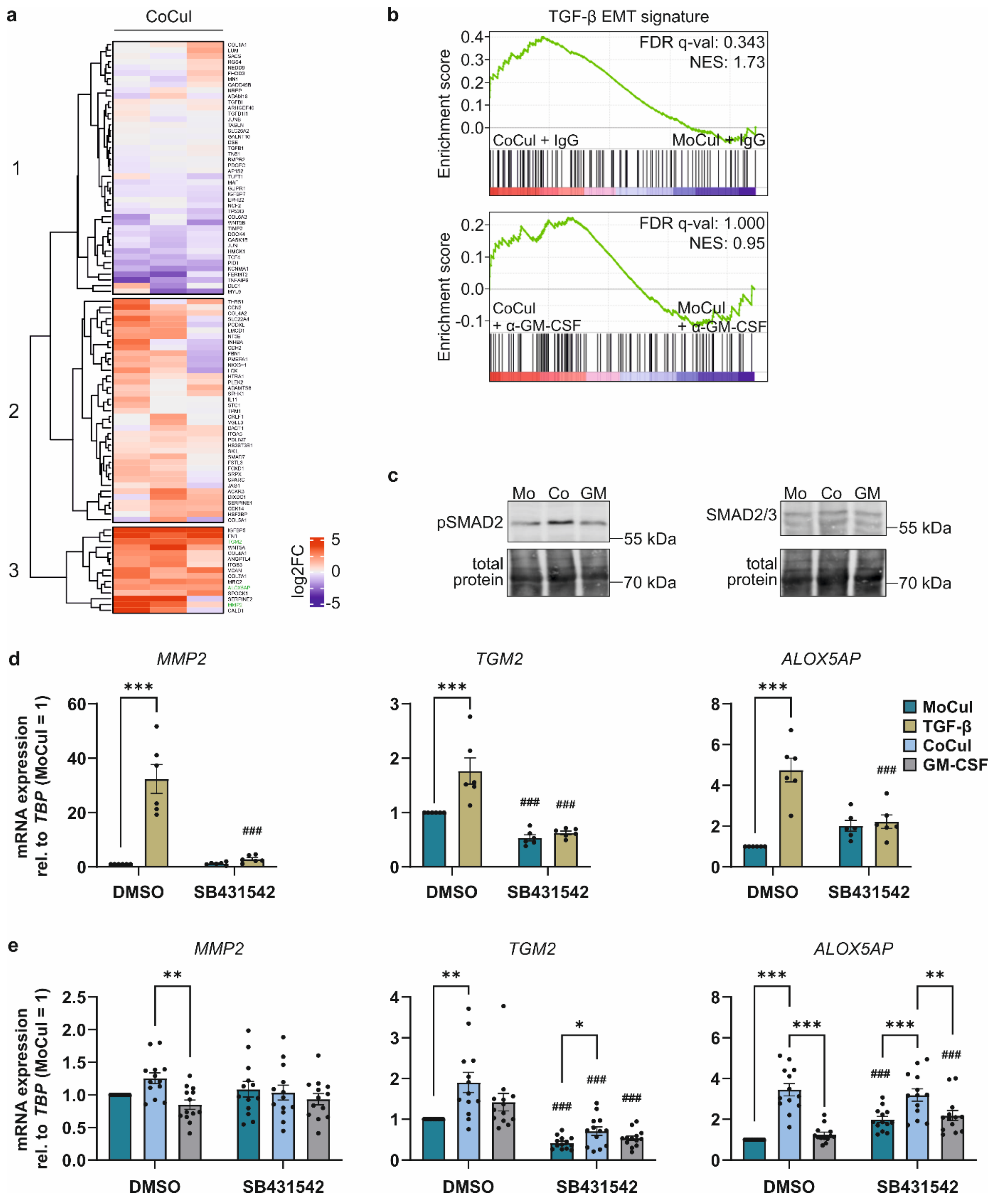

Supplementary Fig. 5: TGF-β signaling is activated in monocytes interacting with GB cells. (**a**) Primary human monocytes were co-cultured with LN229 cells for 48 h. RNA expression was determined by RNA-seq (n = 3). Heatmap visualizes log2FC (co-culture (CoCul) relative to mono-culture (MoCul)) of genes obtained from gene set "(Foroutan) integrated TGF-β EMT-up", categorized by *k*-means clustering. (**b**) Gene Set Enrichment Analysis (GSEA) of mono-cultured (MoCul) vs. co-cultured (CoCul) monocytes treated with polyclonal goat IgG control (IgG) (*upper panel*) or 0.5 µg/mL GM-CSF neutralizing antibody (α-GM-CSF) (*lower panel*) (FDR: false discovery rate; NES: normalized enrichment score). (**c**)

Phosphorylated mothers against decapentaplegic homolog 2 (pSMAD2) protein expression was determined by Western blot analysis and normalized to total SMAD2/3 expression. Blots are representative of 10 independent experiments. (**d**) Primary human monocytes were treated with 1 ng/mL recombinant human transforming growth factor-β (TGF-β, *yellow*) and with an inhibitor for TGF-β signaling (SB431542, 10 μM) for 24 h (n = 6). (**e**) Primary human monocytes were incubated with SB431542 (10 μM) or DMSO, and mono-cultured (MoCul, *cyan*) or co-cultured (CoCul, *blue*) with LN229 cells in transwell inserts, or treated with 50 pg/mL recombinant human granulocyte-macrophage-colony stimulating factor (GM-CSF, *grey*) for 24 h (n = 13). mRNA expression of the TGF-β targets *matrix metalloproteinase 2* (*MMP2*), *transglutaminase 2* (*TGM2*), and *arachidonate 5-lipoxygenase activating protein* (*ALOX5AP*) was analyzed by qPCR and normalized to *TATA-box-binding protein* (*TBP*) expression. All data are means ± SEM and were statistically analyzed by two-way repeated measures ANOVA with Šídák's multiple comparisons test ($^{*}p < 0.05$, $^{**}p < 0.01$, $^{***}p < 0.001$; $^{\#}p < 0.05$, $^{\#\#}p < 0.01$, $^{\#\#\#}p < 0.001$ (compared to respective DMSO controls)).

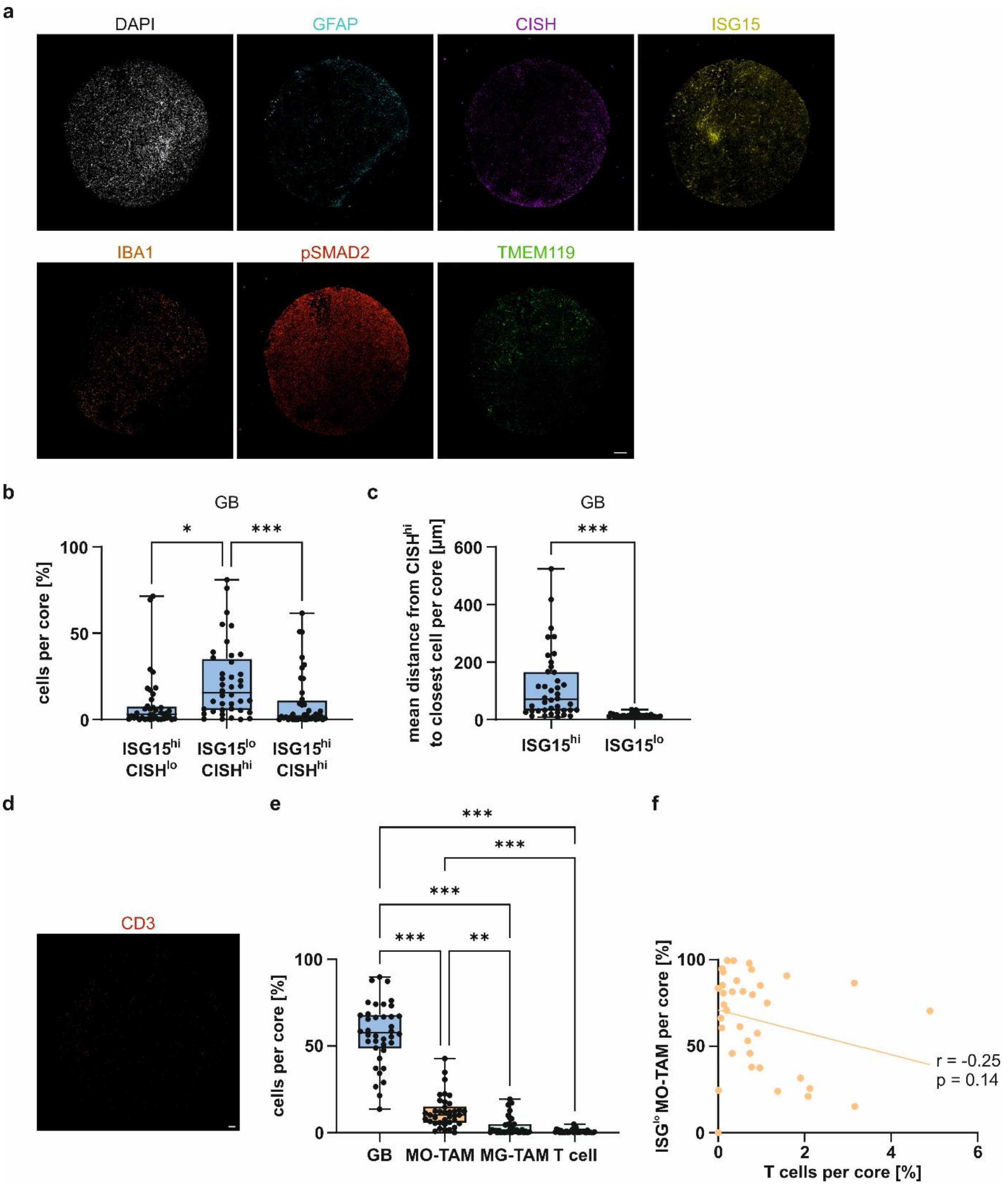

Supplementary Fig. 6: ISG expression in monocyte-derived TAMs negatively correlates with abundant GM-CSF signaling in the GB TME *in vivo*. (**a**) Representative image of one GB core displaying DAPI (*white*), glial fibrillary acidic protein (GFAP, *cyan*), cytokine-inducible SH2-containing protein (CISH, *purple*), interferon-stimulated gene 15 (ISG15, *yellow*), ionized calcium-binding adapter molecule 1 (IBA1, *orange*), phosphorylated mothers against decapentaplegic homolog 2 (pSMAD2, *red*), and transmembrane protein 119 (TMEM119, *green*). Scale bar 20 µm. (**b**) Percentage of cells with high expression of ISG15, or CISH, or both among GB cells. (**c**) Mean minimal distance between $CISH^{hi}$ cells and $ISG15^{hi}$ or $ISG15^{lo}$ tumor cells. (**d**) Representative image of one GB core displaying cluster of differentiation 3

(CD3, *red*). Scale bar 20 µm. (**e**) Percentage of tumor cells and residual resident astrocytes (GB; $GFAP^+$, *blue*), monocyte-derived tumor-associated macrophages (MO-TAM; $IBA1^+TMEM119^-$, *orange*), microglial TAMs (MG-TAM; $IBA1^+TMEM119^+$, *green*), and T cells ($CD3^+$, *red*). All data are presented as boxplots depicting median (line), interquartile range (box) and min/max (whiskers) and were statistically analyzed by Friedman test (**b, e**), or Wilcoxon test (**c**) (*$p < 0.05$, **$p < 0.01$, ***$p < 0.001$). (**f**) Correlation of percentage of ISG^{lo} MO-TAMs and T cells per core. Simple linear regression and calculation of Pearson's correlation coefficient (r) was performed with GraphPad Prism.

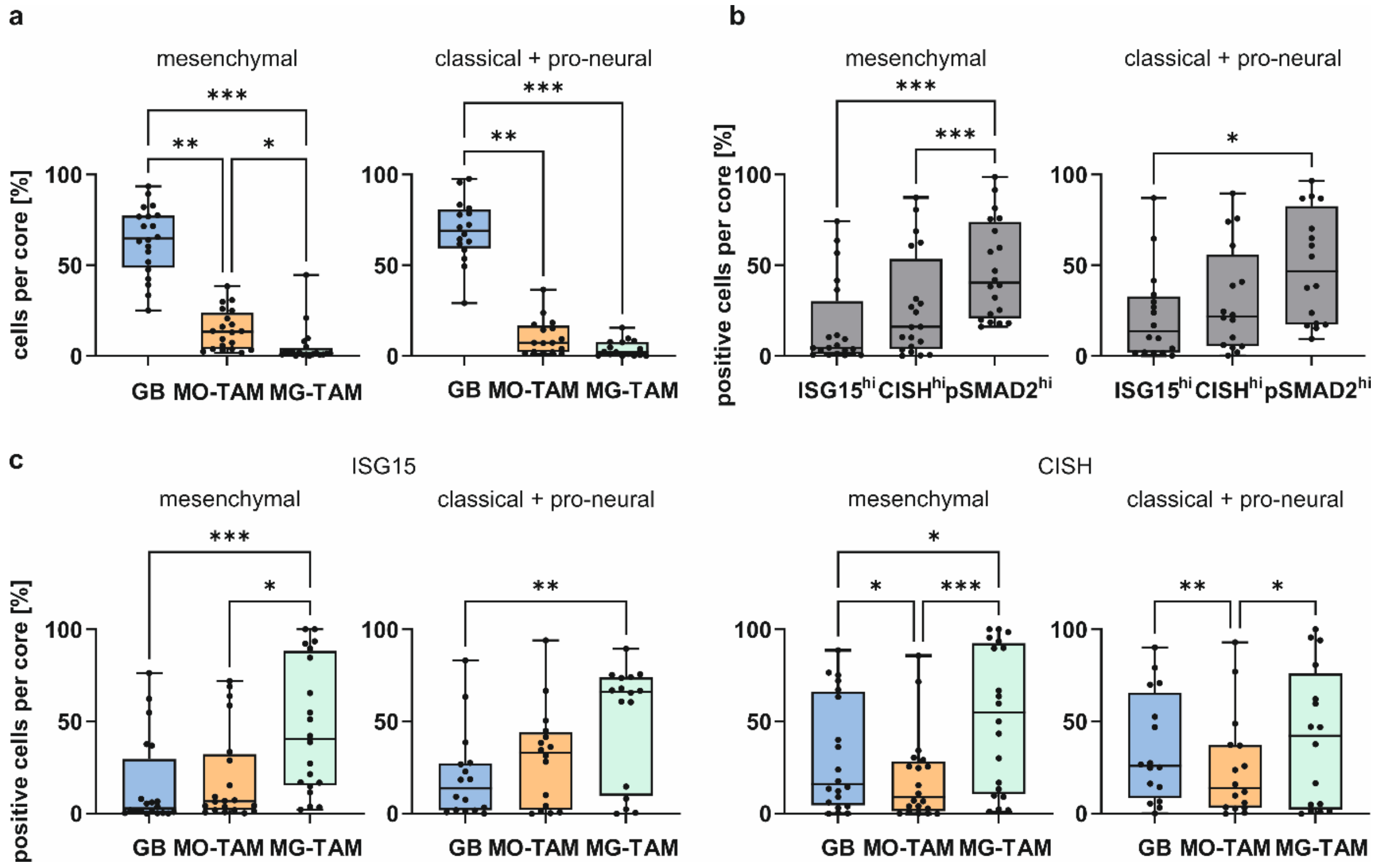

Supplementary Fig. 7: ISG expression is low in monocyte-derived TAMs in the GB TME of the mesenchymal subtype *in vivo.* (**a-c**) GB tissue microarrays from 40 patients from either mesenchymal or classical and pro-neural subtypes were analyzed by multiplex fluorescent immunohistochemistry (mfIHC) using antibodies against glial fibrillary acidic protein (GFAP), ionized calcium-binding adapter molecule 1 (IBA1), transmembrane protein 119 (TMEM119), interferon-stimulated gene 15 (ISG15), cytokine-inducible SH2-containing protein (CISH), and phosphorylated mothers against decapentaplegic homolog 2 (pSMAD2). (**a**) Percentage of tumor cells and residual resident astrocytes (GB; $GFAP^+$, *blue*), monocyte-derived tumor-associated macrophages (MO-TAM; $IBA1^+TMEM119^-$, *orange*), and microglial TAMs (MG-TAM; $IBA1^+TMEM119^+$, *green*), or of (**b**) $ISG15^{hi}$, $CISH^{hi}$, and $pSMAD2^{hi}$ cells per individual core differentiated by mesenchymal vs. classical/pro-neural subtypes. (**c**) Percentage of $ISG15^{hi}$ and $CISH^{hi}$ cells among tumor cells, MO-TAMs, and MG-TAMs differentiated by mesenchymal vs. classical/pro-neural subtypes. All data are presented as boxplots depicting median (line), interquartile range (box) and min/max (whiskers) and were statistically analyzed by Friedman test (*$p < 0.05$, **$p < 0.01$, ***$p < 0.001$).

Supplementary Methods

Bioinformatics analyses of RNA-sequencing and scRNA-seq

During data processing, quality-, adapter-, and polyA-trimming were performed using cutadapt (version 1.18)[2], reads were mapped to the human genome (GRCh38) using STAR (version 2.7.10a)[3], and determination of transcript counts was performed with htseq-count (version 2.0.2)[4]. Differentially expressed genes (DEGs) were determined using the DESeq2 package v1.40.1[5] in R[6] with p values adjusted by Benjamini-Hochberg FDR correction. Visualization with the ComplexHeatmaps package v2.16.0[7] was performed after row-wise *z*-score normalization of read counts or after log2FC normalization of read counts, and transcripts were grouped by *k*-means clustering. For functional annotation, differentially expressed transcripts were analyzed using the Database for Annotation, Visualization and Integrated Discovery (DAVID)[8,9] against the gene set "GOTERM_BP_ALL" with a list of all detected transcripts (rowSums > 4) as background data set. Moreover, statistically enriched biological processes between two conditions were identified with Gene Set Enrichment Analysis (GSEA) (version 4.3.2)[10].

For the analysis of published scRNA-sequencing data (GSM4972211, ND1-7)[1], we used the Seurat package v5.3.0[11] in R. The aggregated count matrix (output from cell ranger) comprising 21,303 cells and 18,774 genes was used as input data. Cells with less than 500 or more than 3,000 detected genes and with more than 15% mitochondrial genes out of total counts were removed from further analyses. Data were normalized and scaled, and variable features were identified using the Seurat function SCTransform. Cells were filtered for CD45 expression (*PTPRC* > 0). Clustering of immune cells was visualized on a two-dimensional map using Uniform Manifold Approximation and Projection (UMAP). A score for the gene signature "hallmark interferon-α response" (MSigDB) was calculated using the UCell package v2.6.2[12] based on Mann-Whitney U statistics.

Bioinformatics analyses of multiplex fluorescent immunohistochemistry (mfIHC) stainings of the tissue microarrays

Unmixed images were generated with the inForm® software V2.3 (Akoya, Marlborough, USA). Overstained edges and folded areas were manually removed in Qupath[13] and cell detection and segmentation were performed using StarDist[14] in Qupath. Individual intensity thresholds of all markers for the whole cell, or nucleus in the case of pSMAD2, were determined for each core based on visual inspection. Tumor cell, MO-TAM, ~~and~~ MG-TAM, and T cell phenotypes were identified based on these individual thresholds. Secondary analysis of detected cells was performed with R. Cells with high (^{hi}) or low (^{lo}) expression of ISG15, CISH, and pSMAD2 were determined based on the median of the respective individual thresholds. Co-localization analysis was performed using the BIOP JaCoP plugin in ImageJ FIJI[15]. Example figures were generated with the QuickFigures plugin[16]. Different molecular subtypes of GB biopsies had

been determined based on DNA methylation profiling and are considered to adequately reflect the TCGA subtypes (mesenchymal = mesenchymal, RTK I = proneural, RTK II = classical)[17].

Statistical analysis

Normal distribution was assessed based on D'Agostino & Pearson test, Anderson-Darling test, Shapiro-Wilk test, and Kolmogorov-Smirnov test. If residuals were assumed not to be normally distributed based on all four tests, data were log-transformed before statistical testing. Statistically significant differences were estimated using either a paired t-test, one-way repeated measures ANOVA with Dunnett’s multiple comparisons test, two-way repeated measures ANOVA with Šídák's multiple comparisons test, or Friedman test, Wilcoxon test or mixed-effects analysis with Šídák’s or Tukey’s multiple comparisons test, if normal distribution did also not apply to log-transformed data.

Supplementary Tables

Supplementary Table 1: Recombinant proteins, neutralizing antibodies, and inhibitors

name	category	manufacturer	catalog number
M-CSF	recombinant protein	Immunotools (Friesoythe, Germany)	11343112
GM-CSF	recombinant protein	Immunotools	11343122
IFN-β1	recombinant protein	Peprotech (Hamburg, Germany)	300-02BC
TGF-β1	recombinant protein	Proteintech (Martinsried, Germany)	HZ-1011
B18R	recombinant protein	ebioscience (Frankfurt, Germany)	34-8185
α-GM-CSF (polyclonal)	neutralizing antibody	biotechne	AF-215-NA
polyclonal normal goat IgG control	neutralizing antibody	biotechne	AB-108-C
α-IFNAR2 (Clone MMHAR-2 Mab)	neutralizing antibody	PBL assay science (Piscataway, NJ, USA)	21385
IgG2a isotype control (Clone C1.18.4)	neutralizing antibody	Biozol (Eching, Germany)	BE0085
SB431542 hydrate	inhibitor	Sigma-Aldrich	S4317

Supplementary Table 2: Antibodies used for fluorescence activated cell sorting (FACS)

target	fluorophore	manufacturer	catalog number
CD45	BUV661	BD Biosciences	750178
CD14	BB700	BD Biosciences	566465
CD16	BV650	BD Biosciences	563692
CD133	BV711	BD Biosciences	747566
IFNAR1	APC	R&D Systems	FAB245A

Supplementary Table 3: Antibodies used for Western blot

target	species	manufacturer	catalog number	blocking
IFIT1	rabbit	Proteintech	23247-1-AP	milk
ISG15	rabbit	Proteintech	15981-1-AP	milk
pSMAD2 (Ser467)	rabbit	Abcam (Cambridge, UK)	ab280888	milk
SMAD2/3	rabbit	Cell Signaling (Frankfurt, Germany)	3102	milk
pSTAT5 (Tyr694)	rabbit	Cell Signaling	9351	milk
STAT5	rabbit	Cell Signaling	94205	milk
pSTAT1 (Tyr701)	rabbit	Cell Signaling	7649S	BSA
STAT1	mouse	Cell Signaling	9172S	BSA

Supplementary Table 4: Primers

primer	forward	reverse
IFIT1	ATTTACAGCAACCATGAGTACAAA	GGCTTCCTCATTCTGGCCTT
OAS3	AGCCCTCCTTTACCAAACCC	GCTGAAGAGCCACCCTTGA
IFI44L	GACTTCTCAAAGCCGGGTCAT	GGGCTGTAACGCTCTCTCAA
ISG15	CAGCGAACTCATCTTTGCCAG	GACACCTGGAATTCGTTGCC
IFI6	CCATCTATCAGCAGGCTCCG	CACCCCACTGCAAGTGAAGA
HERC5	GTGGAAAATGACTGTGGACGC	AGATCAAACGTGGGCCTCAA
CISH	CTCTGCGTTCAGGGGTGAG	TGGAGCGCAGCAGTCTAAAA
IFNAR1	CCGTTTGTCATTTATGCTGCG	CAATGGCTGTTCAGAGAAATACTCA
MMP2	AGGGCACATCCTATGACAGC	ATTTGTTGCCCAGGAAAGTG
TGM2	GGCACCAAGTACCTGCTCA	AGAGGATGCAAAGAGGAACG
ALOX5AP	TCAGCGTGGTCCAGAATGG	GCAAGTGTTCCGGTCCTCT

Supplementary Table 5: Antibodies used for multiplex fluorescent immunohistochemistry (mfIHC)

target	species	manufacturer	catalog number
GFAP	mouse	Proteintech	60190-1-Ig
TMEM119	rabbit	Sigma-Aldrich	HPA051870
IBA1	rabbit	Fujifilm (Ratingen, Germany)	019-19741
ISG15	rabbit	Proteintech	15981-1-AP
pSMAD2	rabbit	Abcam (Cambridge, UK)	ab280888
CISH	rabbit	Abcam	ab191447

References

1. Pombo Antunes, A. R. *et al.* Single-cell profiling of myeloid cells in glioblastoma across species and disease stage reveals macrophage competition and specialization. *Nat. Neurosci.* **24**, 595–610 (2021).
2. Martin, M. Cutadapt removes adapter sequences from high-throughput sequencing reads. *EMBnet.journal* **17**, 10 (2011).
3. Dobin, A. *et al.* STAR: ultrafast universal RNA-seq aligner. *Bioinformatics* **29**, 15–21 (2013).
4. Anders, S., Pyl, P. T. & Huber, W. HTSeq--a Python framework to work with high-throughput sequencing data. *Bioinformatics* **31**, 166–169 (2015).
5. Love, M. I., Huber, W. & Anders, S. Moderated estimation of fold change and dispersion for RNA-seq data with DESeq2. *Genome Biol.* **15**, (2014).
6. R Core Team. R: A Language and Environment for Statistical Computing. R Foundation for Statistical Computing (2021).
7. Gu, Z., Eils, R. & Schlesner, M. Complex heatmaps reveal patterns and correlations in multidimensional genomic data. *Bioinformatics* **32**, 2847–2849 (2016).
8. Huang, D. W., Sherman, B. T. & Lempicki, R. A. Systematic and integrative analysis of large gene lists using DAVID bioinformatics resources. *Nat. Protoc.* **4**, 44–57 (2009).
9. Sherman, B. T. *et al.* DAVID: a web server for functional enrichment analysis and functional annotation of gene lists (2021 update). *Nucleic Acids Res.* **50**, W216–W221 (2022).
10. Subramanian, A. *et al.* Gene set enrichment analysis: A knowledge-based approach for interpreting genome-wide expression profiles. *Proc. Natl. Acad. Sci.* **102**, 15545–15550 (2005).
11. Hao, Y. *et al.* Dictionary learning for integrative, multimodal and scalable single-cell analysis. *Nat. Biotechnol.* **42**, 293–304 (2024).
12. Andreatta, M. & Carmona, S. J. UCell: Robust and scalable single-cell gene signature scoring. *Comput. Struct. Biotechnol. J.* **19**, 3796–3798 (2021).
13. Bankhead, P. *et al.* QuPath: Open source software for digital pathology image analysis. *Sci. Rep.* **7**, 16878 (2017).
14. Schmidt, U., Weigert, M., Broaddus, C. & Myers, G. Cell Detection with Star-Convex Polygons. in *Medical Image Computing and Computer Assisted Intervention – MICCAI 2018* (eds. Frangi, A. F., Schnabel, J. A., Davatzikos, C., Alberola-López, C. & Fichtinger, G.) vol. 11071 265–273 (Springer International Publishing, Cham, 2018).
15. Schindelin, J. *et al.* Fiji: an open-source platform for biological-image analysis. *Nat. Methods* **9**, 676–682 (2012).
16. Mazo, G. QuickFigures: A toolkit and ImageJ PlugIn to quickly transform microscope images into scientific figures. *PLOS ONE* **16**, e0240280 (2021).
17. Capper, D. *et al.* DNA methylation-based classification of central nervous system tumours. *Nature* **555**, 469–474 (2018).
